# Across the Gap: Geochronological and Sedimentological Analyses from the Late Pleistocene-Holocene Sequence of Goda Buticha, Southeastern Ethiopia

**DOI:** 10.1371/journal.pone.0169418

**Published:** 2017-01-26

**Authors:** Chantal Tribolo, Asfawossen Asrat, Jean-Jacques Bahain, Cécile Chapon, Eric Douville, Carole Fragnol, Marion Hernandez, Erella Hovers, Alice Leplongeon, Loïc Martin, David Pleurdeau, Osbjorn Pearson, Simon Puaud, Zelalem Assefa

**Affiliations:** 1 IRAMAT-CRP2A, UMR 5060, CNRS, Université de Bordeaux, Pessac, France; 2 School of Earth Sciences, Addis Ababa University, Addis Ababa, Ethiopia; 3 Laboratoire « Histoire naturelle de l'Homme préhistorique », UMR 7194, CNRS, Département de Préhistoire du Muséum National d'Histoire Naturelle, Paris, France; 4 Laboratoire des Sciences du Climat et de l’Environnement, UMR 8212, CEA-CNRS, Gif-sur-Yvette, France; 5 Institute of Archaeology, Hebrew University, Jerusalem, Israel; 6 Institute of Human Origins, Arizona State University, Phoenix, United States of America; 7 McDonald Institute for Archaeological Research, Cambridge University, Cambridge, United Kingdom; 8 Department of Anthropology, University of New Mexico, Albuquerque, United States of America; 9 Department of Anthropology, Archaeobiology Program & Human Origins Program, National Museum of Natural History, Washington, D.C., United States of America; Universidade do Algarve, PORTUGAL

## Abstract

Goda Buticha is a cave site near Dire Dawa in southeastern Ethiopia that contains an archaeological sequence sampling the late Pleistocene and Holocene of the region. The sedimentary sequence displays complex cultural, chronological and sedimentological histories that seem incongruent with one another. A first set of radiocarbon ages suggested a long sedimentological gap from the end of Marine Isotopic Stage (MIS) 3 to the mid-Holocene. Macroscopic observations suggest that the main sedimentological change does not coincide with the chronostratigraphic hiatus. The cultural sequence shows technological continuity with a late persistence of artifacts that are usually attributed to the Middle Stone Age into the younger parts of the stratigraphic sequence, yet become increasingly associated with lithic artifacts typically related to the Later Stone Age. While not a unique case, this combination of features is unusual in the Horn of Africa. In order to evaluate the possible implications of these observations, sedimentological analyses combined with optically stimulated luminescence (OSL) were conducted. The OSL data now extend the radiocarbon chronology up to 63 ± 7 ka; they also confirm the existence of the chronological gap between 24.8 ± 2.6 ka and 7.5 ± 0.3 ka. The sedimentological analyses suggest that the origin and mode of deposition were largely similar throughout the whole sequence, although the anthropic and faunal activities increased in the younger levels. Regional climatic records are used to support the sedimentological observations and interpretations. We discuss the implications of the sedimentological and dating analyses for understanding cultural processes in the region.

## 1-Introduction

A large body of recent and ongoing archaeological researches in sub-Saharan Africa has focused on the transition between the Middle Stone Age (MSA) and the Later Stone Age (LSA), e.g. at Border cave [1], Mumba Rock Shelter [2], and Enkapune Ya Muto [3]. These studies have extended the date of the earliest LSA occurrences back to ca. 50 ka BP. Typically the start of LSA is marked by the appearance of more complex hunting and fishing technologies (hafted microliths, bows and arrows, polished bone tools) or symbolic items (e.g., ostrich eggshell or marine shell beads, engraved ocher, eggshells, bones and wood [4–6]), even though some of these items appeared earlier during the MSA (e.g., during the Stillbay and Howiesons Poort in southern Africa [7–9]). These studies indicate that the emergence of the LSA may involve variable technical, symbolic or social aspects in different geographic regions and different points in time. The mosaic pattern might be partly related to human dispersals and demographic shifts within and out of Africa, assumed to occur from ca. 70 ka onward, e.g. [1,10–13].

**Fig 1:**
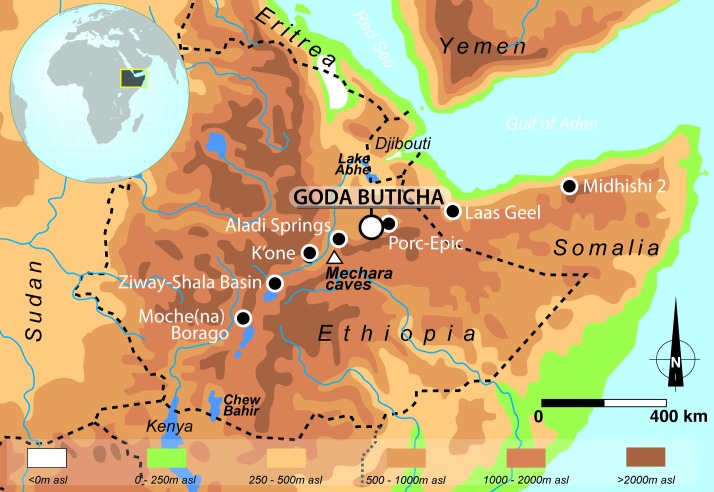
The Horn of Africa with location of Goda Buticha and sites mentioned in the text.

In the Horn of Africa, the evidence and processes of the MSA-LSA transition are poorly understood. Indeed, despite a history of extensive archaeological surveys [14–19], comparatively few stratified sites document this period. In Ethiopia, such sites include K’one [20], Porc-Epic [21–23], Aladi Springs [16, 24, 25], Ziway-Shala Basin sites [26], and Mochena Borago [27]. In Somalia, the site of Midhishi 2 also falls within this time range [28, 29], (Fig 1). In most of these sites, the archaeological sequences are short, discontinuous, and/or not well dated. At Mochena Borago and the Ziway-Shala Basin sites, which record a relatively long time span from Marine Isotope Stage (MIS) 4–3 to the Holocene, the sequences seem to share a major chronostratigraphic gap broadly corresponding to MIS 2 [26, 27].

A similar gap has been observed at Goda Buticha in southeastern Ethiopia [30]. The archaeological stratigraphic sequence was first dated with radiocarbon from ~ 46 ka cal BP for the lower layers, to ~ 1.5 ka cal BP near the present-day surface level. The dates revealed a chronological gap, from ~ 33 ka cal BP (end of MIS 3) to ~ 7.8 ka cal BP (mid-Holocene) in the sedimentary sequence [30] (see §2.2 for details). The cultural assemblages of Goda Buticha do not show a clear technological rupture before and after the gap. The lowest layers show MSA technical characteristics with few LSA components, while the upper layers contain typical MSA components, together with artefacts with clear LSA characteristics (see §2.2). The consistency of the radiocarbon ages, which display no significant chronostratigraphic inversion, and the absence of clear evidence for large-scale disturbances of the stratigraphic layers such as redeposition by cut and fill, argue against post-depositional mixing as an explanatory mechanism for this pattern. The late persistence of MSA components is unusual (though not unknown) in the region [30, 31], and required corroboration through sedimentological and dating analyses that allow better control for temporal and depositional processes.

Recently, genetic studies have shown the important role of the Horn of Africa in Upper Pleistocene and Holocene human dispersals from and into Africa [32–36]. There have also been suggestions that certain areas in the Horn of Africa, specifically the Southwest High Plateau, served as biogeographic refugia to Upper Pleistocene populations during times of climatic instability or deterioration [27, 37]. Such scenarios are important when we evaluate the sequence from Goda Buticha and its place within the larger picture of human history on the African continent.

The main goals of this study were i) to extend the age model of the stratigraphic sequence by dating the lowermost levels, where no charcoal could be dated; ii) to test the robustness of the ^14^C chronology by using an additional dating technique, and in particular to confirm or refute the existence of the chronological gap; iii) if confirmed, to investigate how the gap is manifested in the sedimentological and geochemical site formation processes. In this paper, we present detailed sedimentological analyses combined with a new chronological framework based on optically stimulated luminescence ages (OSL) on sediments from the various stratigraphic units of Goda Buticha.

## 2-The Goda Buticha (Buticha Cave) sequence

### 2.1 Site setting

**Fig 2:**
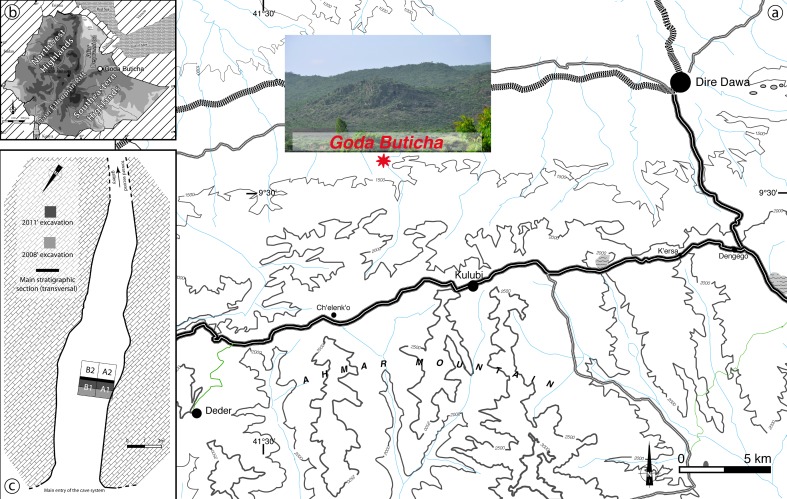
**Map of the Goda Buticha area (a), view of the escarpment where the cave is located (b) and map of the cave showing the excavated area (c)**.

In 2007, geological and archaeological survey of cave formations in eastern Ethiopia led to the discovery of several previously unknown or poorly known caves bearing anthropogenic deposits [38]. Among them, Goda Buticha (“Cave of the Ghost”), revealed a deep archaeological sequence, comprising multiple Paleolithic occupation horizons [30]. The cave, situated ~15 km west of the city of Dire Dawa at an elevation of 1382 m above mean sea level (asl), is located in the escarpment of the Southeastern Ethiopian Highlands to the south, just outside the Main Ethiopian Rift to the north (Fig 2). It is carved into Jurassic crystalline limestone beds in a cliff side and comprises two main galleries oriented NNW and NE, connected by a short perpendicular passage. The sedimentological sequence is deposited in the NNW gallery of the branching cave system [38].

We thank the Ethiopian Authority for Research and Conservation of Cultural Heritage (ARCCH) for authorization to excavate in Goda Buticha. The excavations at Goda Buticha took place in 2008 and 2011. At a distance of four meters from the cave entrance two square-meters were dug inside the cave, revealing a > 230 cm thick sequence (Fig 2). More than 5 m^3^ of sediments have been removed and sieved, and several thousand archaeological finds (lithics, faunal remains, and pottery) have been plotted in 3D or recovered from a 2 mm-mesh sieve. Some of the archaeological materials and their general chronostratigraphic context have been studied and published elsewhere [30, 31, 38, 39, 40]. In the following section, we summarize the main features and results.

### 2.2 Stratigraphy, ^14^C chronology and cultural sequence at Goda Buticha

**Fig 3:**
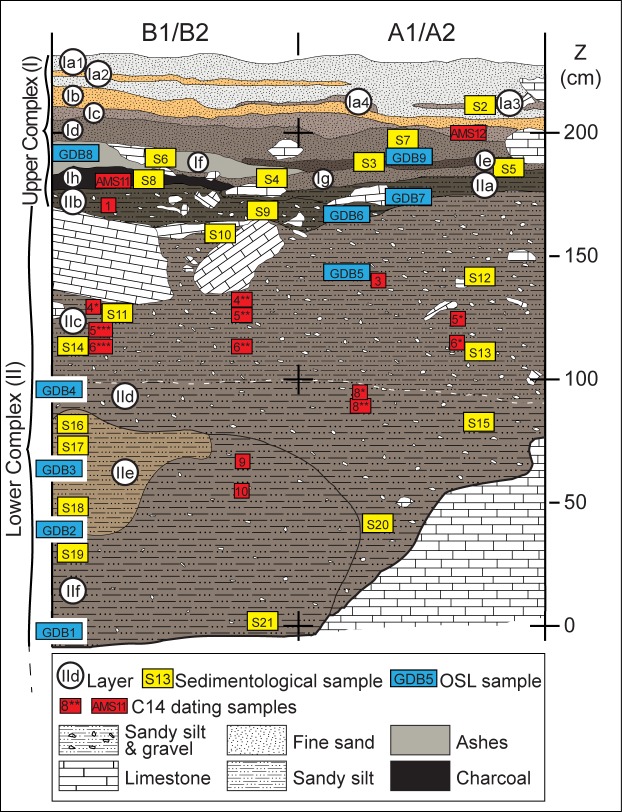
Stratigraphic section of the Goda Buticha sequence. The locations of the OSL samples, ^14^C samples, and samples for sedimentological analyses are shown. Note that the nomenclature has changed for the layers of Complex I compared to previous publications [30].

The excavated stratigraphic sequence of Goda Buticha site is 230 cm thick, but the bedrock has not been reached. The top is partially capped by deposits of modern bat guano. The sequence is divided into two distinct complexes [30] (Fig 3 and S1 Fig): Complex II (also named “Lower Complex” in previous publications) at the bottom (at elevation of 0 to 180 cm above datum), and Complex I (also “Upper Complex” in earlier publications) at the top (180 to 230 cm above datum). The sediments are dry and powdery in both complexes. Two sedimentological subsets were identified in Complex II: (i) from Layer IIf to Layer IId, the sediment is silty and contains limestone clasts. In Layer IIf, the sediment is partially impregnated by calcium carbonate; (ii) from Layer IIc to Layer IIa, the sediment also has a silty matrix but the limestone fragments are more numerous than in the lower sub-unit and of more diverse sizes, ranging from gravels to blocks. The contact between the two subsets is only weakly distinguished in the field by this difference in limestone concentration and size, but there is no marked discontinuity.

Complex I is formed by a succession of ashy layers (Layers Ih to Ia1). The sediments are dark in color, sandy to silty, with a high calcium carbonate content and abundant micromammals remains. They are devoid of a coarse fraction.

Fourteen AMS radiocarbon analyses on charcoal samples [30], have provided ages for Complex II (Table 1): (i) the lower sub-unit of Complex II is of Upper Pleistocene age, dating from at least 46273 ± 2084 cal BP (42500 ± 1000 BP, top of Layer IIf) to 33843 ± 405 cal BP (29680 ± 230 BP, Layer IId), thus falling within MIS 3; (ii) the upper sub-unit of Complex II is of a Holocene age with dates from 7791 ± 126 cal BP (6920 ± 50 BP, Layer IIc) to 4676 ± 144 cal BP (4130 ± 30 BP, Layer IIb). Two additional charcoal samples have provided consistent late Holocene ages for Complex I, of 4060 ± 90 cal BP (3725 ± 30 BP, Layer If) and 1485±75 cal BP (1610 ± 30 BP, Layer Ic). Note that there is some scatter in the ^14^C ages and thus there are a few chronostratigraphic reversals (within Layer IIf and within Layer IIc), but there is no sign of significant mixing between the samples of Pleistocene and Holocene age, or between the samples of mid and late Holocene.

**Table 1:** Radiocarbon ages for charcoal samples from Goda Buticha.

Complex	Elevation above datum (cm)	Layer	Grid square	Lab référence	Number in Fig 3	Uncalibrated age	Calibrated Age BP
**Complex I**	200–210	Ic	A1a/A1b	SacA#42484	12	1610 ± 30	**1487 ± 47**
	190–180	If	B1a/B1b	SacA#42483	11	3725 ± 30	**4067 ± 55**
**Complex II upper sub-unit**	180–170						
	170–160	IIb	B1c/B1d	SacA#27480	1	4130 ± 30	**4680 ± 81**
	160–150						
	150–140	IIc	A1c/A1d	SacA#27479	3	6410 ± 30	**7351 ± 43**
	140–130	IIc	B1a/B1b	SacA#35205	4**	6200 ± 30	**7094 ± 57**
	140–130	IIc	B1c/B1d	beta #255124	4*	5590 ± 50	**6372 ± 47**
	130–120	IIc	A1a/A1b	beta#255123	5*	6920 ± 50	**7757 ± 56**
	130–120	IIc	B1a/B1b	beta#269596	5**	6940 ± 40	**7771 ± 51**
	130–120	IIc	B1c/B1d	beta#269597	5***	6430 ± 40	**7357 ± 42**
	120–110	IIc	A1a/A1b	beta#269594	6*	6470 ± 40	**7376 ± 40**
	120–110	IIc	B1a/B1b	beta#269595	6**	6370 ± 50	**7314 ± 60**
	120–110	IIc	B1c/B1d	beta#255122	6***	6440 ± 50	**7360 ± 46**
**Complex II lower sub-unit**	110–100						** **
	100–90	IId	A1c/A1d	beta#269593	8*	29680 ± 230	**33839 ± 198**
	100–90	IId	A1c/A1d	beta#255121	8**	31590 ± 270	**35486 ± 306**
	90–80						** **
	80–70						** **
	70–60	IIf		SacA#35207	9	42500 ± 1000	**46089 ± 1042**
	60–50	IIf		SacA#35206	10	38870 ± 670	**42907 ± 525**

All the ^14^C samples are charcoals. The 95% confidence interval is indicated for all data. The ^14^C ages have been calibrated using Oxcal and the Intcal13 calibration curve [41, 42].

**Fig 4:**
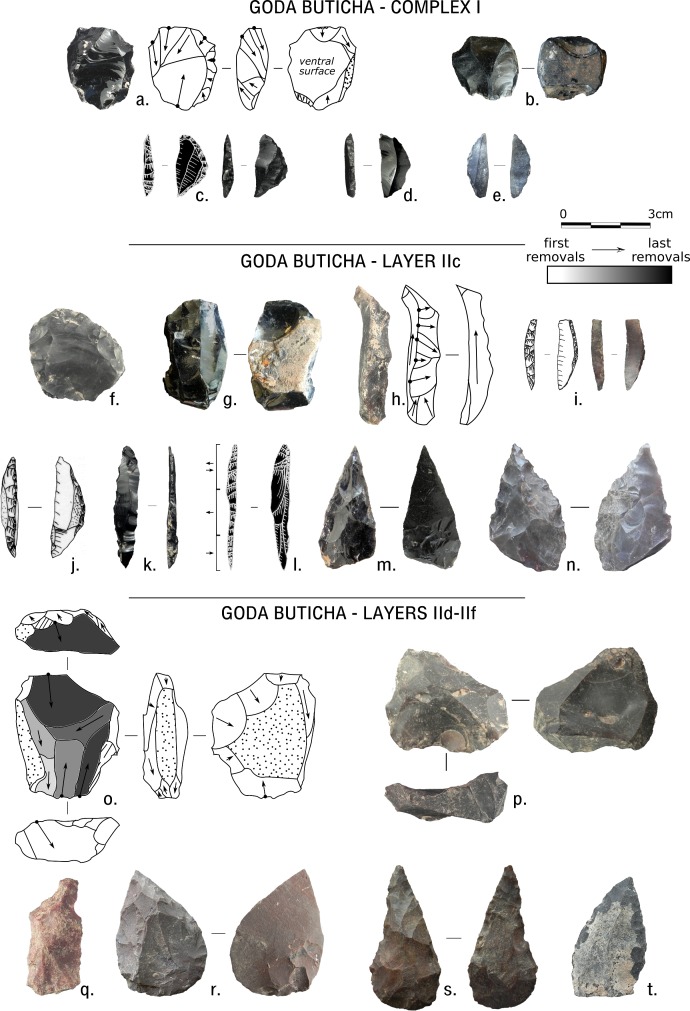
Lithic artifacts from Complex I and Layers IIc and IId-IIf of Goda Buticha. Complex I: a, b: cores; c-e: geometric microliths. Layer IIc: f: Levallois preferential core; g: blade core; h: crested blade; i, j: backed pieces, i is microlithic; k, l: elongated backed points; m, n: bifacial retouched points. Layers IId-IIf: o,p: cores; p is Levallois-like preferential core; q: Levallois blade; r,s,t: retouched points.

The dates suggest a chronological and stratigraphic gap within Complex II. This gap does not coincide with the main visible change in the sediments (which is between Complex II and Complex I) or with any abrupt cultural change [30]. The lower sub-unit of Complex II (Layers IId-IIf) shows clear MSA technical features, with few LSA components (Fig 4). The assemblage is oriented towards flake production together with relatively high frequencies of items related to blade production and with typical MSA unifacial and bifacial points. Both flakes and blades were obtained through the use of a planimetric conception of debitage, including Levallois methods, with typical MSA unifacial and bifacial points. The upper sub-unit (Layers IIa-IIc) shows a clear tendency for the production of blades, bladelets, and non-geometric microliths in tandem with typical MSA components (e.g. unifacial or bifacial points and Levallois products [31, 40]). Complex I, despite a very low density of artifacts, is characterized by very different technical features, such as the abundant use of obsidian to produce small flakes and blades that were then transformed into geometric microliths [30, 39].

A small sample (n = 340) of mammalian faunal remains were piece-plotted during the excavation. Several hundreds of small fragments of medium to large-sized mammals, as yet unstudied, were collected from the sieved sediments. Of the piece-plotted faunal remains, 10% are teeth or jaws, 10% are long bone shaft fragments, and the rest (80%) are unidentifiable bone fragments. Many of the remains are in friable condition, with poor surface preservation, which discouraged initial efforts to identify any anthropogenic or carnivore modifications. Thus, the preliminary analysis focused on taxonomic identifications [30].

A fairly diverse set of taxa has been identified, including Procaviidae, Lagomorpha, *Equus*, *Phacochoerus*, a small carnivore, and several types of bovids including bovini, antilopini, *Redunca*, and *Tragelaphus*. No domesticated fauna were recovered from any of the stratigraphic levels at the site. Nearly 100 fragments of ostrich eggshell (some measuring up to 4–5 cm in diameter) occurred throughout the sediment column, with frequencies increasing from the bottom to the top of the sequence. Likewise, micro-faunal remains, sometimes heavily burned, occur throughout the sequence, but their relative frequency and the proportion of burned specimens both decrease with depth. A detailed analysis of the microfauna and its paleoenvironmental implication will be published elsewhere, so the data will not be discussed here.

In addition to the rich archaeological assemblages, the excavation yielded ten hominin specimens [30]. A cranial vault fragment was found in Complex II (Layer IId), at an elevation of 40 cm above datum and is potentially older than ~42–46 ka cal BP (Table 1). The human remains of Goda Buticha, recovered from the MIS 3 layers (i.e. Layer IId, close to the 47–42 ka ^14^C cal BP samples) and from the Holocene deposits (i.e., Layer IIc dated from ca. 7 to 4 ka cal BP) are of importance given the paucity of human fossils from the time range of MIS 3 to 1 [43] and given the genetic and archaeological arguments for out of Africa and back to Africa movements during the Upper Pleistocene and Holocene [33, 35, 36].

## 3- Materials and Methods

To obtain the goals of this research regarding the chronology of the site and its depositional dynamics, the entire stratigraphic sequence was sampled at high resolution (Fig 3). Nine samples were analyzed for OSL dating at the Institut de Recherche sur les ArchéoMATériaux (IRAMAT), University of Bordeaux-Montaigne. Each of the nine OSL samples were divided into two sub-samples, one of which was added to the separate batch of sixteen sediment samples exported to the Museum National d’Histoire Naturelle (MNHN) in Paris for sedimentological and geochemical analyses.

### 3.1 Sedimentological analyses

After dry sieving through a 2 mm mesh in the lab, each sample was split into several aliquots. The first one was used for quantification of carbonates using a calcimeter and organic material by calcination at 375°C for 16 hours. The second aliquot was used for measurement of pH and grain size analyses. After wet sieving and drying, the 50–200 μm fraction was observed under an optical microscope for the determination of heavy and light minerals and for the analysis of the quartz grain morphoscopy. Magnetic susceptibility was measured at low frequency (0.47 kHz) for each bulk sample. Another portion of each bulk sample was powdered and used for X Ray Diffraction (XRD) analyses. Mineralogical identifications and semi-quantitative estimates were then performed. Further details about the analytical procedures are given in Appendix 1.

### 3.2 Optically stimulated luminescence (OSL) dating analyses

Luminescence is a paleodosimetric dating method. In order to calculate ages, two main parameters must be determined: the total dose accumulated by the samples since the dated event (equivalent dose, De) and the dose delivered by the environment per unit of time (dose rate, Dr). The dated event is the last exposure to sunlight (or of heating to high temperature), which resets the OSL signal. The methods for estimating the De rely on the measurements of luminescence signals, the intensity of which is related to the amount of dose received since the last reset [44, 45]. We describe here the main procedures of the analyses that were conducted for the determination of the De and Dr. Details about the preparation of the samples, measurement units and tests of the protocols for the De estimate can be found in Appendix 2. Comments specific to the beta Dr estimation are provided in Appendix 3.

Collection of the nine sediment samples, labeled GDB1 to 9 (Fig 3), was conducted at night, using subdued orange light, and the samples were immediately packed into black plastic bags. In the laboratory, quartz grains in the size range of 200–250 μm were extracted from each sample following standard procedures (Appendix 2). Tests for the protocols of the De determinations were conducted on both multi-grains and single-grain aliquots, while for the final De measurements single-grain aliquots were preferred since they avoid any averaging effects. The single aliquot and regenerative dose protocol was applied (SAR [46, 47]; Appendix 2).

The total Dr can be decomposed into cosmic, gamma, alpha and beta Dr. The cosmic Dr were calculated by applying the equation of Prescott and Hutton [48], taking into account the geometry of the cave ceiling. The gamma Dr were estimated from field gamma spectrometry (using a LaBr_3_(Ce) 1.5x1.5 probe, Canberra), conducted using the threshold technique [49, 50]. The alpha Dr were assumed to be negligible since the external part of the grains had been etched. The beta Dr was estimated from the U, Th and K contents of each sample following Martin [51, 52]. This protocol intends to investigate the effect of heterogeneity at the millimeter scale on the Dr estimate (Appendix 3). After drying, fine grinding and sealing of the samples, U, Th and K contents of the fine (< 2 mm) and coarse (2 mm-1 cm) fractions were analyzed separately with high-resolution gamma spectrometry (HRGS), except for the coarse fraction of samples GDB3, 4, 7 and 8. For these sub-samples, Inductively Coupled Plasma Spectrometry (ICP-MS and ICP-AES) was used instead, because the prepared amount of each sub-sample was too small for standard HGRS. The beta Dr was then calculated using the attenuation factors for the grain size of Guérin et al. [53] and specific dose rates of Guérin et al. [50]. Water content (see §4.2.1) was incorporated into dose rate calculations to account for attenuation.

## 4- Results

### 4.1 Sedimentology

**Fig 5:**
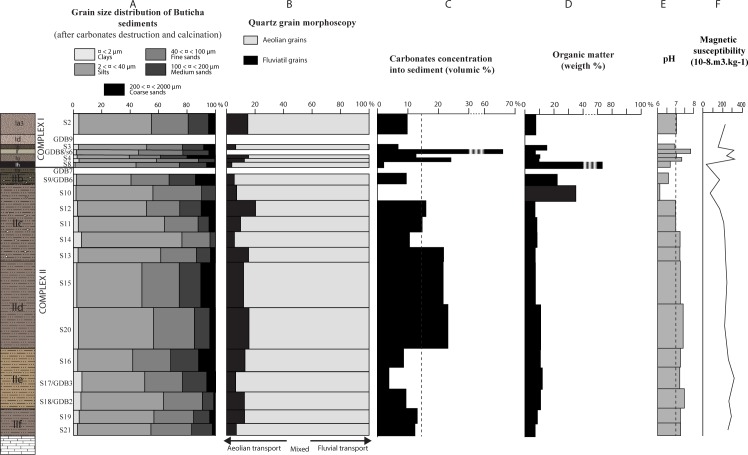
Synthesis of the sedimentological analyses of the Goda Buticha sediments. A: grain size fractions of decarbonated sediments; B: quartz grains morphoscopy; C: carbonate content; D: organic matter content; E: pH values; F: magnetic susceptibility.

**Fig 6:**
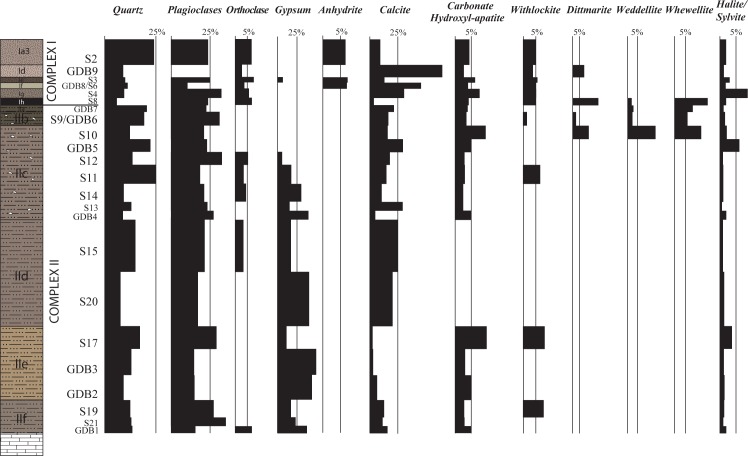
Mineralogy of the Goda Buticha sediment based on X-ray diffraction analyses. Numbers are provided in S1 Table. The percentages are relative to peak heights in the diffractogram. As the sensitivities of the XRD signal differs for each mineral, they can only be considered as semi-quantitative results.

The results of the geological analyses are displayed in Fig 5 (grain size analyses, quartz grain morphoscopy, carbonate and organic quantification, pH values, and magnetic susceptibility) and in Fig 6 and S1 Table (XRD analyses).

Twenty mineralogical species corresponding to 6 chemical groups were identified by XRD: magmatic minerals (quartz, feldspars, muscovite, chlorite, hornblend, rutile), carbonates (calcite, dolomite), evaporites (gypsum, anhydrite, halite, sylvite), phosphates (carbonate hydroxyl-apatite, dittmarite, whitlockite), calcium oxalates (weddellite, whewellite) and clays (kaolinite, montmorillonite). The minerals (determined on sieved and decarbonated sediment) are dominated by magmatic minerals (25 to 55%) and are present in all samples. Calcite (determined from the raw sediment) is abundant (5 to 70%) and present in the entire sequence; it dominates in the lower part of Complex I (Layers Ig/If), and the middle part of Complex II (Layer IId).

#### Complex II

Complex II is characterized by brown, well-sorted silts and fine sands (Fig 5A). The clay concentrations are low (< 5%) but omnipresent (Fig 5A). The morphoscopy analyses show that on average, 80% of the quartz grains are sub-rounded and lusterless while 20% are angular or sub-rounded and shiny (Fig 5B). The variation of the morphoscopic classes through Complex II is not statistically significant. In addition to quartz and feldspar (plagioclase and orthoclase) grains, apatite and a limited variety of heavy minerals including green hornblend, zircon and sphene are observed. Calcium carbonates are more abundant in Layer IId (~22% in volume) than in the other layers of Complex II (~5 to 10%) (Fig 5C), but the limestone clasts are more frequent in the upper part (IIc). Halite and sylvite occur in low quantities (~2%) in all layers. Calcium phosphates (carbonate hydroxyl-apatite, whitlockite and dittmarite) are present at the bottom (Layers IIe, IIf) and at the top (Layer IIc) of Complex II. The alternating presence of gypsum and calcium oxalates (weddellite and whewellite) divides the infilling into two parts below and above Z~150 cm, i.e. from IIf to middle of IIc and from middle of IIc to IIa (Fig 6, S1 Table).

Organic matter is present in all layers (though it was macroscopically observed only in some of them) (Fig 5D) and its percentage (~10% in weight) remains constant through Complex II up to Z~150 cm. In the upper part of Complex II (above Z~150 cm, i.e. top of Layer IIc to Layer IIa), the organic content is significantly higher, up to 34%. This is likely because microfaunal remains and seeds are more frequent than in the lower part (Fig 5D). The top of Layer IIc (sample S10) in particular corresponds essentially to a dark brown accumulation of biogenic elements including fragments of insect carapaces and tiny balls that can be attributed to bat metabolic wastes. Throughout the stratigraphy, the pH is neutral or slightly basic (pH between 7 and 7.5) but becomes slightly acidic (pH 6.2) at the top of Complex II, above Z~150 cm (Fig 5E). The magnetic susceptibility values (Fig 5F) are nearly constant through Complex II, slightly decreasing in its upper layers, above Z~150 cm (top of IIc and IIb).

#### Complex I

Layer Ih differs markedly from the other layers of Complex I and is described separately. The other Complex I layers are characterized by moderately well sorted silts and fine sands (Fig 5A). The sediments consist mainly of ashes and become richer in microfaunal bones in the upper levels. The mineralogical content (quartz, feldspar and heavy minerals) and the quartz grain morphoscopy are close to, but more variable than, those of Complex II. Calcite is present in all these layers and is particularly abundant in Layers Ig to If (31 to 76%, S1 Table). Evaporites are present in the form of gypsum (only in Ie), anhydrite (in Ie, Id, Ia3), halite and sylvite (in all these layers). Calcium phosphates are found over the whole Complex I except dittmarite, which is present only in If and Ih.

The dark grey Layer Ih (sample S8) shows significantly high organic matter content (~75% in weight) associated with abundant charcoal fragments (< 1 mm), burned insect carapaces, and microfaunal remains (Fig 5D). The sorting of silts and fine sand in Ih is better than that of other layers in Complex I. In this layer, the quartz grains are sub-rounded and lusterless (Fig 5B). Moreover, Ih differs by its paucity in calcite, and the presence of dittmarite and Ca-oxalates, both nearly absent in the other layers of Complex I.

The pH is neutral but slightly acidic in Layer Ih (6.7) and slightly basic in Layer Ig (7.25) and If (7.8). The magnetic susceptibility values are more variable in Complex I than in Complex II (Fig 5F), ranging from 37 to 320 10^−8^ m^3^/kg.

### 4.2 Optically stimulated luminescence dating analyses

#### 4.2.1 Dose rate stability over time and beta dose rates

In this section we first discuss the reliability of the Dr (i.e. whether the current Dr can be representative of the past mean Dr), based on the examination of the U, Th and K contents. Once we have established that the current Dr is meaningful for dating purposes, we discuss the accuracy of the beta Dr estimate, bearing in mind that the heterogeneity of the sediments at a millimetric scale must be considered (Appendix 3).

**Fig 7:**
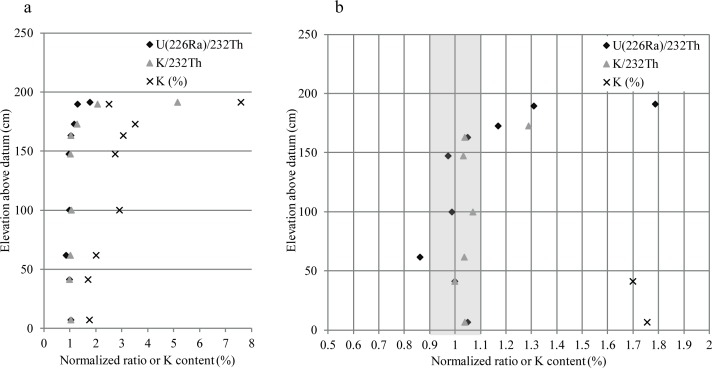
K content and ^226^Ra/^232^Th and K/^232^Th normalized ratio plotted against the sample elevation. The ratios are normalized to the contents of sample GDB2. Fig 7b is a higher resolution view of the data in Fig 7a.

U, Th and K contents are presented in Table 2A, 2B and 2C for the fine (< 2 mm), coarse (2 mm– 1 cm) and total (< 1 cm) fractions, respectively. For the fine fraction, the most striking pattern is the high K content, ranging between 1.7 and 7.6% and reaching its highest values in the upper layers of Complex I. The K content can be related to the presence of orthoclase, sylvite and illite. Its contribution to the total Dr can be roughly estimated to range between 75 and 92%. It is therefore important to ensure that the K content has been stable over the burial time. We argue here that this was likely the case in Complex II. Firstly, because of the presence of soluble minerals (gypsum and sylvite), which suggests negligible percolation: water dripping, leading to the crystallization of gypsum, was quickly followed by evaporation. Sylvite crystallizes directly from bat urine and cannot be associated with waterlogged conditions. As the water content is currently low (0–6% in Table 3), it can be safely assumed that it was also the case during the deposition of the sediments coeval to the formation of the evaporites. Moreover, there is no strong evidence for diagenetic processes that would be expected if significant percolation had occurred in such an environment (e.g. [54]). A second argument for the stability of the K content over time is the nearly constant ratio of K/^232^Th and ^226^Ra/^232^Th, at elevation from 7 cm to 163 cm above datum (Fig 7): ^232^Th is not soluble while ^40^K and part of the U chain are. The consistency of the ratio is likely not fortuitous and suggests that conditions were relatively similar during the period of sediment deposition into the cave. The change of this ratio above 163 cm is likely attributable either to a change in the nature of the sediments (Complex I versus Complex II) and/or to additional input of K and U. This is further discussed in section 4.2.3 and section 5.

**Table 2:** U, Th and K contents.

a	sample GDB	^238^U (ppm)						^232^Th (ppm)		K (%)	
		(^234^Th)		(^226^ Ra)		(^210^Pb)					
	9	1.28	±0.10	1.64	±0.03	1.17	±0.16	2.23	±0.09	7.595	±0.056
	8	0.68	±0.04	0.99	±0.01	1.09	±0.10	1.84	±0.06	2.523	±0.023
	7	1.91	±0.14	1.99	±0.04	1.72	±0.13	4.14	±0.20	3.527	±0.029
	6	2.26	±0.22	1.93	±0.05	1.66	±0.16	4.47	±0.27	3.065	±0.031
	5	2.25	±0.16	1.61	±0.03	1.27	±0.12	4.03	±0.19	2.747	±0.024
	4	2.06	±0.16	1.68	±0.03	1.17	±0.12	4.13	±0.20	2.913	±0.026
	3	1.40	±0.08	1.04	±0.01	0.89	±0.10	2.94	±0.11	2.011	±0.020
	2	1.30	±0.07	1.06	±0.01	0.81	±0.09	2.58	±0.08	1.700	±0.016
	1	1.07	±0.07	1.11	±0.02	1.02	±0.11	2.56	±0.10	1.756	±0.020
b	sample GDB	^238^U (ppm)						^232^Th (ppm)		K (%)	
		(^234^Th)		(^226^ Ra)		(^210^Pb)					
	9	1.57	±0.11	1.36	±0.02	0.85	±0.22	1.49	±0.06	5.247	±0.067
	8	1.25	±0.04	nd		nd		2.05	±0.04	1.975	±0.101
	7	1.72	±0.09	nd		nd		1.86	±0.07	3.497	±0.178
	6	2.02	±0.12	1.47	±0.03	1.46	±0.22	1.65	±0.07	1.143	±0.021
	5	1.31	±0.15	0.80	±0.03	0.80	±0.29	1.14	±0.08	0.846	±0.022
	4	0.83	±0.02	nd		nd		0.50	±0.00	0.840	±0.043
	3	0.69	±0.01	nd		nd		1.52	±0.01	1.040	±0.053
	2	2.17	±0.14	1.25	±0.03	1.02	±0.28	1.32	±0.08	0.881	±0.022
	1	0.88	±0.10	0.63	±0.02	0.45	±0.20	1.66	±0.06	1.103	±0.022
c	sample GDB	mass proportion of coarse fraction		^238^U (ppm)				^232^Th (ppm)		K (%)	
				(^234^Th)		(^226^ Ra)					
	9	22%		1.46	±0.11	1.57	±0.03	2.02	±0.07	6.944	±0.047
	8	16%		0.77	±0.03			1.87	±0.05	2.436	±0.025
	7	6%		1.88	±0.12			3.71	±0.16	3.521	±0.041
	6	13%		2.18	±0.19	1.84	±0.04	4.07	±0.24	2.750	±0.027
	5	13%		1.88	±0.13	1.29	±0.02	2.89	±0.12	1.994	±0.017
	4	4%		2.00	±0.15			3.97	±0.19	2.821	±0.025
	3	7%		1.35	±0.08			2.84	±0.10	1.942	±0.019
	2	12%		1.41	±0.07	1.08	±0.01	2.42	±0.07	1.598	±0.014
	1	20%		1.01	±0.05	0.96	±0.01	2.28	±0.08	1.547	±0.015

a) U, Th and K contents for the < 2 mm fraction deduced from high resolution gamma spectrometry. The U contents are deduced from the activity of different daughters in the decay chain: mainly ^234^Th, ^214^ Pb and ^214^Bi in equilibrium with ^226^Ra, and ^210^Pb. b) U, Th and K contents for the 2 mm-1 cm fraction deduced from high resolution gamma spectrometry (GDB1, 2, 5, 6, 9) or ICP-MS or AES (GDB3, 4, 7, 8). c) Mean U, Th and K contents calculated from a and b, weighted by the mass proportion of the < 2 mm and 2 mm-1 cm fractions. The proportion of the 2 mm– 1 cm fraction per sample is indicated (i.e. mass of 2 mm-1 cm fraction on total dry mass of material < 1 cm) These content values represent the contents that would have been obtained if the whole < 1 cm fraction had been crushed, homogenized and analyzed.

**Table 3:** Dose rates for each sample.

sample GDB	Complex	current water content	dose rate (Gy/ka)													
			gamma		cosmics		beta-1		total -1		beta-2		total -2		total- mean	
9	Complex I	6%	0.814	±0.087	0.088	±0.011	5.350	±0.691	6.252	±0.697	5.000	±0.589	5.902	±0.594	6.077	±0.639
8		0%	1.072	±0.123	0.088	±0.011	1.962	±0.249	3.122	±0.278	1.913	±0.216	3.073	±0.243	3.098	±0.259
7		3%	0.704	±0.078	0.088	±0.011	2.783	±0.340	3.575	±0.349	2.778	±0.305	3.570	±0.314	3.572	±0.330
6	Complex II upper sub-unit	2%	0.748	±0.083	0.088	±0.011	2.492	±0.300	3.328	±0.312	2.299	±0.245	3.135	±0.257	3.231	±0.280
5		5%	0.527	±0.057	0.088	±0.011	2.162	±0.258	2.777	±0.265	1.979	±0.211	2.594	±0.218	2.686	±0.238
4	Complex II lower sub-unit	4%	0.527	±0.057	0.088	±0.011	2.301	±0.277	2.916	±0.283	2.228	±0.241	2.843	±0.247	2.879	±0.263
3		4%	0.588	±0.149	0.088	±0.011	1.593	±0.192	2.269	±0.243	1.538	±0.167	2.214	±0.222	2.241	±0.232
2		4%	0.507	±0.055	0.088	±0.011	1.356	±0.162	1.951	±0.171	1.289	±0.136	1.884	±0.146	1.918	±0.157
1		6%	0.535	±0.057	0.088	±0.011	1.354	±0.162	1.977	±0.172	1.255	±0.135	1.878	±0.145	1.927	±0.157

The uncertainties include both statistic and systematic errors. Beta-1 and total-1 refer to the beta and total dose rates calculated from the U, Th and K contents of the < 2 mm material, and -2 refers to the beta and total dose rates calculated from the whole sediment U, Th, K contents (excluding > 1 cm material). Their mean has been used for the final age calculations.

It can also be noted that the U chain is slightly in disequilibrium (e.g., pre-^226^Ra/post-^226^Ra activities ratio from -39 to +31% of unity) (S2 Table). However, the impact of this disequilibrium might have on the total Dr over time is almost negligible (U contribution 4–13% compared to the contribution of the K (75–92%) to the total Dr).

The U, Th and K contents in the coarse fraction are significantly different from those of the fine fraction (e.g., K content for the coarse fraction of GDB5 is 69% lower than for the fine fraction—Table 2B). The beta Dr were calculated separately from the mean U, Th and K contents of the fine material (contents in Table 2A and dose rate beta-1 in Table 3) and from the whole material < 1 cm (contents in Table 2C and dose rates beta-2 in Table 3). It is expected on theoretical grounds that the true beta Dr falls between these two estimates. In order to confirm this and evaluate which beta Dr was closest to the true beta Dr, simulations were carried out (Appendix 3). It was observed that the true beta Dr always ranges between the beta-1 Dr and the beta-2 Dr in the different cases that were investigated. Consequently, a mean value should be the best option to estimate the beta Dr for the samples. However, the calculations show that for these samples, the fine (beta-1 and total-1) and fine plus coarse (beta-2 and total-2) Dr are relatively close to each other (ratio of total-1 to total-2 < 1.07) and statistically indistinguishable. Therefore, the correction calculated by this approach remains small for the Goda Buticha samples: the large differences in radio-isotope content is counterbalanced by the low relative weight of the coarse fraction (4 to 22% of the total, Table 2C) and the contribution of other components of the Dr (gamma and cosmics).

The total Dr ranges between 1.92 ± 0.16 Gy/ka to 3.57 ± 0.33 Gy/ka for GBD1 to 8 and up to 6.08 ± 0.64 Gy/ka for GDB9 (Table 3).

#### 4.2.2. Equivalent dose distributions

The reliability of the SAR protocol for the GDB samples in this study was tested on multi-grain aliquots with dose recovery tests and preheat plateau tests. Dose recovery tests were also conducted on single grains, prior to final single-grain De measurements that were used for age determination. The tests and their results are described in Appendix 2.

**Fig 8:**
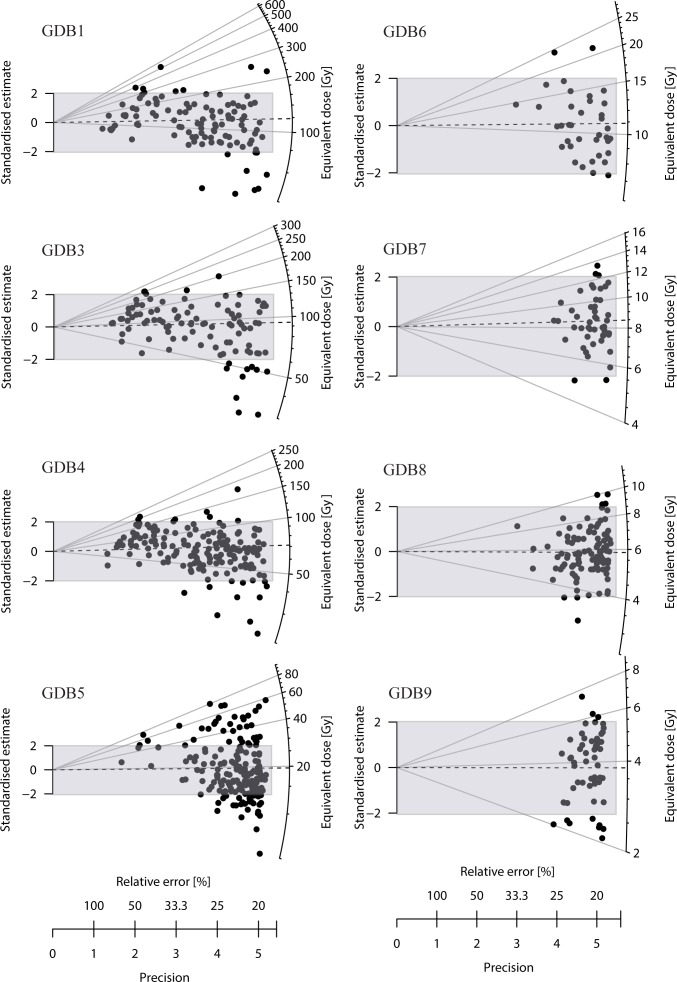
Radial plots for the De distributions of each sample. The number of measured and accepted grains, central De (CAM) and over-dispersion can be found in Table 4. The over-dispersion due to the measurement, estimated to be at least 19% has been added quadratically to the relative uncertainty. The shaded region emphases the central De ± 2 sigma.

**Fig 9:**
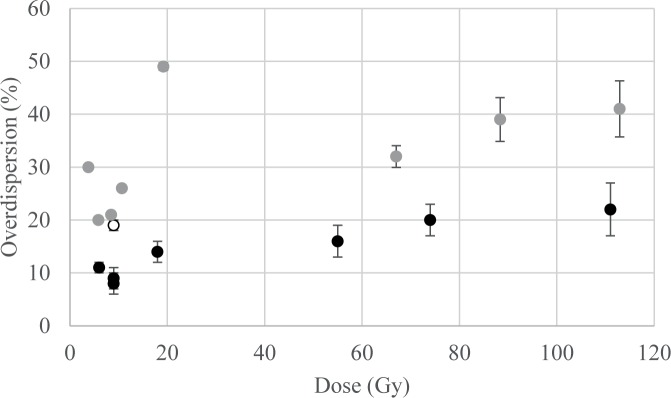
Over-dispersion in function of the dose. Black dots: beta dose given inside the reader (dose recovery test); white dot: gamma dose given outside the reader; grey dots: natural dose. A longer explanation of the OD of the beta and gamma dosed samples is given in Appendix 2.

Single-grain D_e_ distributions are plotted in Fig 8. The central D_e_ (after the central age model—CAM—of [55]) increases significantly from top to bottom, from 3.8 ± 0.2 Gy to 112.9 ± 5.3 Gy (Table 4), and the over-dispersions (OD—i.e. dispersion of the De beyond the statistical dispersion expected in a lognormal distribution) increase with De, from 20 ± 2% to 41 ± 4% except for sample GDB9 (30 ± 3%) and 5 (49 ± 3%) (Fig 9).

**Table 4:** Results of the De analyses.

sample GDB	preheat parameters		n	overdispersion (%)		De (Gy)					
	natural and regenerative dose	test dose				CAM		unweighted arithmetic mean		bayesian central	
9	260°C 10s	160 cut	55/200	30	±3	3.8	±0.2	4.0	±0.2	4.0	±0.2
8	260°C 10s	240 cut	100/400	20	±2	5.9	±0.1	6.0	±0.1	5.9	±0.1
7	260°C 10s	220 10s	47/400	21	±3	8.5	±0.3	8.7	±0.3	8.7	±0.3
6	260°C 10s	220 10s	38/300	26	±4	10.7	±0.5	11.4	±0.6	10.8	±0.5
5	260°C 10s	220 10s	188/1100	49	±3	19.2	±0.7	22.7	±1.0	18.4	±0.6
4	260°C 10s	220 10s	168/800	32	±3	67.0	±2.0	77.5	±2.6	71.3	±1.7
3	260°C 10s	160 cut	100/700	39	±4	88.4	±4.1	104	±4.7	96.6	±4.1
1	260°C 10s	160 cut	106/700	41	±4	112.9	±5.3	134	±7.3	121.9	±5.1

The uncertainties include the counting uncertainty and the systematic uncertainty due to the reproducibility of the illumination. The number “n” of accepted/measured single grains is indicated. The De are calculated using the CAM [55], or unweighted mean [56] or Bayesian central [57] models.

The interpretation of the De distributions, which reflect the pre- and post-deposition history of the quartz grains, is not straightforward, the main question being whether the distribution is consistent with a single “mean” age or presents multiple components, due to poor bleaching or bioturbation. In Appendix 2 we suggest that–based on the geological data, on the comparison between single and multi-grain De_s_, and on the comparison of the natural and artificial (beta or gamma dose given in the laboratory) OD–the De distributions in this study are consistent with single dose components.

For the final calculation of the relevant “mean” De from each De distribution, several models are currently discussed in the literature: the widely used central age model (CAM) of Galbraith et al. [55], the recently developed Bayesian model of Combès et al. [57] and the simple, non-weighted arithmetic mean advocated by Guérin et al. [56]. In Table 4, the three estimates are indicated for each sample. Presentation of a detailed comparison of the three models is beyond the scope of this paper. It can simply be noted that they are statistically indistinguishable for the youngest samples (GDB 9, 8, 7 and 6). For the older samples, the variations between the estimates increase slightly, but the Bayesian estimate is in most cases consistent at one or two sigma with the CAM or arithmetic mean (except for GDB5 when compared to the unweighted mean, and for GDB4 when compared to the CAM, though in this last case, the discrepancy is low- <6%). Since the Bayesian SAR model is statistically more robust and allows a better treatment of the error estimates [57], it has been used for the age calculations and comparison with the ^14^C data. The Bayesian De range between 4.0 ± 0.2 Gy and 121.9 ± 5.1 Gy.

#### 4.2.3. OSL ages and comparison with the ^14^C chronology

**Fig 10:**
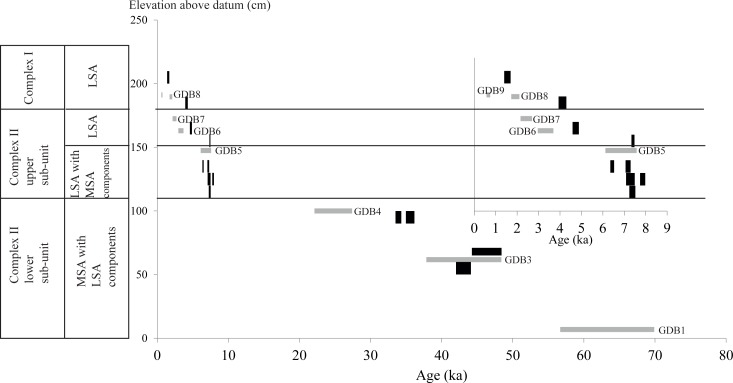
Plots of age estimates as a function of the elevation within the stratigraphy. The intervals represent the confidence interval at 95%. Black: ^14^C data; grey: OSL data. The inset zooms in the < 10 ka data.

The OSL age estimates (Table 5) are shown in Fig 10 together with the ^14^C calibrated ages (Table 1). The OSL ages extend the chronostratigraphy of the sequence to the start of MIS 3 or end of MIS 4 (63 ± 7 ka). The age of 43 ± 5 ka for sample GDB3 is consistent with the two ^14^C ages, though those were in apparent chronostratigraphic reversal. The chronological gap within Complex II at ~105 cm above datum that was suggested by the ^14^C dates is confirmed by the OSL results, albeit it is now of a shorter duration (between 24.8 ± 2.6 ka–OSL—and 7.5 ka cal BP - ^14^C). The age for sample GDB5 at the base of Layer IIa of Complex II (6.9 ± 0.7 ka) is also in agreement with the ^14^C date. The general agreement between the ^14^C and OSL data for Complex II, from Layer IIf up to the top of Layer IIc, argues both against any post-depositional mixing and against any Dr instability.

**Table 5:** OSL ages.

sample GDB	Complex	Layer	Dose rate (Gy/ka)		De (Gy)		Age (ka)	
9	Complex I	Ie	6.077	±0.639	4.0	±0.2	0.7	±0.1
8		Ia	3.098	±0.259	5.9	±0.1	1.9	±0.2
7		IIa	3.572	±0.330	8.7	±0.3	2.4	±0.3
6	Complex II upper sub-unit	IIb	3.231	±0.280	10.8	±0.5	3.3	±0.4
5		IIc	2.686	±0.238	18.4	±0.6	6.9	±0.7
4	Complex II lower sub-unit	IIb/IIc	2.879	±0.263	71.3	±1.7	24.8	±2.6
3		IIe	2.241	±0.232	96.6	±4.1	43	±5
2		IIe/IIf	1.918	±0.157	nd		nd	
1		IIf	1.927	±0.157	121.9	±5.1	63	±7

The ages are calculated using the Bayesian approach developed by Combès et al. (2015) (Table 4) and the dose rates presented on Table 3.

For the top of Complex II (sample GDB6) and all of Complex I, the two chronologies differ. The OSL ages of 3.3 ± 0.4 and 0.7 ± 0.1 ka are younger than the ^14^C ages of 4680 ± 81 cal BP and 1485 ± 75 cal BP, respectively. A possible explanation would be a late input of potassium in bat urine from the current surface. In such a case, the current Dr would be overestimated compared to the past mean Dr, leading to underestimate of the calculated OSL ages. For GDB6, though the K/^232^Th ratio is similar to those for Complex II, suggesting beta Dr stability, the proximity (< 30 cm) to the upper most layers, receiving a late input of K, would imply increased gamma Dr.

## 5- Discussion

### 5.1 Interpretations of the sedimentological data

Analyses of the sediments provide information about the mode of deposition and post-depositional evolution of sediments in Goda Buticha. There is no clear indication of an erosional surface where the chronostratigraphic hiatus appears, suggesting an absence of deposition rather than post-depositional erosion. The consistency of the magnetic susceptibility from the bottom of Complex II up to its upper part suggests the absence of pedogenesis, as do the absence of pedofeatures such as CaCO_3_ redistribution, clay illuviation, organic matter concentration or pH variations.

The consistency of the magnetic susceptibility from the bottom of Complex II up to its upper part also suggests a constant main source for the sediments, and a deposition under highly similar conditions, irrespective of the significant chronological hiatus in the sediment sequence. This is further supported by the other analyses. Over all, the sedimentological analyses show the association of four modes of sedimentation:

**(i) A ubiquitous allochtonous geogenic sedimentation.** Well sorted silts and the dominance of sub-rounded and lusterless quartz grains are attributed to aeolian dusts. The low but omnipresent clay concentrations could indicate the persistence of run-off inputs into the cave, though they could also be due to *in situ* alteration of feldspars. The mineralogical associations of this geogenic fraction of the sediments are characteristic of granitic or clastic sedimentary rocks: the magmatic minerals (silicates and oxides, including heavy minerals) could derive from erosion of Precambrian gneissic, granitic and migmatitic basement rocks that form the ridges above the tectonically downfaulted sedimentary sequence of the Dire Dawa area [58]. Another possible origin is the Triassic and Cretaceous sandstones, which are present in the Harrar plateau and exposed in the immediate vicinity of the site [59, 60]. These rocks have been continuously eroded by streams flowing from the plateau into the rift, accumulating thick alluvial fans and sheetwash deposits in the extended rift basin in front of the cave opening.

**(ii) An autochthonous geogenic sedimentation,** characterized by limestone clasts within Complex II layers and calcite matrix: the limestone in which the cave formed [61] is likely the local source for those.

**(iii) An ubiquitous biogenic sediment** composed of numerous micro-vertebrates, seeds and insect remains. In particular in Complex I, the anthropic and biologic activities could be the main reason for the moderate sorting of the sediments, due to trampling effects.

**(iv) A secondary chemical formation** during and after deposition as attested by mineralogical associations through XRD analyses. The presence of calcium phosphates, evaporites (halite, sylvite and gypsum) and calcium oxalate minerals could be linked to the accumulation of bat guano and urine, which leached and reacted with the calcium carbonates (limestone of the cave) at relatively high temperatures and thus at high evaporation rates [62–64]. In particular, Ca-oxalates, which appear in Complex I, can be related to bat guano and to human-made fires that burned wood or other plants. It is likely that the chemically unstable Ca-oxalates formed in vegetal ashes (see for example [65]) produced calcite by CO_2_ absorption (e.g. in Layers Ia). Evaporitic halite and sylvite are observed in all layers while gypsum, which is produced by the reaction of urine on limestone, is present in the lower part of Complex II up to Z~150cm. In the upper layers of Complex I, gypsum is replaced by anhydrite, another likely consequence of fires in the cave: heat leads to dehydration of gypsum or to evaporation of saline solutions such as bat urines and thus to the formation of anhydrite [63].

In summary, the sedimentation in Goda Buticha cave was mainly induced by aeolian action in both Complex II and Complex I, with a small contribution of run-off. This is consistent with the absence of typical features or morphologies that could be linked to sedimentary dynamics of fluvial origin, like cross beddings, positive grading, laminations, channels and erosive contact. The geological analyses show that the origin and mode of deposition before and after the chronological gap are identical but, in Complex I, anthropic and faunal activities are higher.

It is possible to interpret the results of the geological analyses in terms of environmental conditions in the cave’s environments during the time of deposition. The conservation of very soluble minerals (such as gypsum, halite, and sylvite) in the lower part of the sequence (Layer IId-IIf) is a good indicator of negligible water circulation (at least vertically) into the sediments, and indicate a local low-moisture regime during and after the sedimentation of the lower part of the sequence. Moreover, the small sample of identifiable fauna contains some open woodland taxa suggesting ecotone conditions including a source of water that could support grasslands.

Regional climate records display a complex picture, though there are few relevant records for the MIS 3 and MIS 2 periods. The period 50–15 ka is poorly represented in the paleoclimate literature of the region. Those few paleoclimatic sequences that are available are mostly from the Northwest Ethiopian Highlands, which belong to a different climate regime (different moisture source), making comparison with the Goda Buticha site complicated. Better records are available for the African Humid Period. Though some earlier ^14^C dated records from Lake Abhe (e.g., [66, 67]) indicate relatively moist conditions during the period 50–31 ka, recent, well-constrained and higher resolution data from the Ethiopian rift suggest intermediate moisture conditions interrupted by drier periods between ~50–35 ka, more pronounced aridity between 35–19 ka, and increasingly moist conditions since then, especially during the African Humid Period (~ 15–5 ka BP), and finally arid conditions during the Late Holocene [68, 69]. Stable isotope records on speleothems from the Mechara area, some 150 km to the southwest of Goda Buticha, show warm and wet conditions in the SE Ethiopian plateau during the Early to Middle Holocene followed by dry conditions during the Late Holocene [70–72].

Therefore, the chronostratigraphic hiatus at Goda Buticha coincides temporally first with the main part of the dry period that started at around 35 ka, then with the African Humid Period and the Early Holocene wet phase. The aeolian activity, which was the main transport agent throughout the sequence, apparently occurred during both wet and dry phases. It is therefore likely that the sedimentological parameters at Goda Buticha did not linearly or immediately respond to the regional climatic shifts. In particular, the regional climate record suggests that the aeolian deposition does not necessarily correspond to the driest phases.

### 5.2 Implications for human occupation

The association in Goda Buticha of late MSA technological characteristics with LSA components during the mid-Holocene is unusual in the Horn of Africa [30]. However, it has already been described for Gobedra [73] and for the lower units of Laas Geel shelter 7 [74], dated to the Terminal Pleistocene. The new chronological and sedimentological data argue against post-depositional mixing as an explanation for the presence of MSA characteristics in the upper levels, where LSA technological characteristics become more frequent. The data confirm that in Goda Buticha the Holocene MSA characteristics post-date a gap of some 17,500 years (from ~25 to ~ 7.5 ka) in the depositional sequence.

The earlier part of the temporal gap in the Goda Buticha sequence coincides with MIS 2, which was arid [68, 69]. Continuity of human occupation may not have been possible in this area during that time. As no archaeological data are currently available in the Horn of Africa for the period corresponding to MIS 2, local persistence of technological traditions currently seems an unlikely explanation for the Holocene MSA traits in the upper part of the sequence [31]. Reintroduction of cultural traits or technological convergence are possible alternative explanations for the MSA-like technical features of Layer IIc, but they cannot be tested regionally [75].

Only a few sites in the Horn of Africa have yielded contemporaneous Late Pleistocene (Mochena Borago, Porc-Epic or Ziway-Shala Basin) and/or Holocene occupations (Mochena Borago, Ziway-Shala Basin). The emerging regional variability of the lithic assemblages, with shared technical components as well as specificities, may derive from differences in their specific geographic locations and local environmental conditions, influences of resource availability, subsistence strategies, and mobility patterns. Regional-scale population movements within Africa (from possible refugia areas) also may have acted as a trigger for converging and/or recurring technological behaviours leading to mosaic patterns of cultural variability in the region [31, 69, 76].

## 6- Conclusion

The OSL dating of the whole sequence of Goda Buticha confirms and refines the previous chronostratigraphy that was based on C14 dating [30]. In particular, the results highlight a chronological and sedimentological hiatus from ca 25 to 7.5 ka cal BP. However, the depositional hiatus is hardly visible through the sedimentological analyses, except for an increase in limestone fragments after the gap. Aeolian transport has remain the main transport agent, irrespective of regional climate changes, while the sedimentary hiatus, was coeval first to very arid conditions, then to wet phases. The relationship between the sedimentary sequence and regional / local climate variability remains to be studied in detail through on-site proxies for climate change, for example micromorphological work and a systematic study of the ubiquitous micromammals in the sequence.

Our current results validate previous observations that an assemblage with both MSA and LSA technological features post-dates the hiatus. These results bear on the high technical variability of the Late Pleistocene and Holocene in the Horn of Africa and emphasize the complexity of the shift from industries labelled “Middle Stone Age” to those labelled “Late Stone Age”.

## Appendix 1: protocols for geological analyses

Each sediment sample was split into several aliquots. After dry sieving at 2 mm, two ~ 8 cm^3^ aliquots were retained for subsequent analyses. The first aliquot was decarbonated with 10% diluted hydrochloric acid. At the end of the reaction, the sample was washed and centrifuged several times to eliminate the acidic solution. It was then mixed in distilled water by magnetic stirring for 4 hours. In the final stage of the stirring, and without stopping, a small volume of sediment was sampled using a needle with a 2 mm opening and analyzed with a magnetometer. The magnetic susceptibility of each sample was determined at low frequency (0.47 kHz) using the Bartington Susceptibility Meter model (MS2B). Air measurements were made before and after each sediment sample analysis. Results presented in Fig 5F are the average of 3 successive measurements.

The grain-size distribution was determined on portions of the first aliquot before and after the decarbonation. It was measured with a laser Mastersizer 2000 granulometer, which estimates the mean diameters of the particles within a size range of 0.02 to 2000 μm.

Another portion of the decarbonated aliquot was used to measure organic matter. It was weighted, carbonized continuously in a muffle furnace at 375°C for 16 hours and weighted again. The loss of weight is attributed to the burning of the organic matter.

The pH value was determined on the second aliquot. Twenty ml of sediment were mixed with 20 ml of distilled water and stirred for four hours. Then the pH of the sediment was measured by plunging a pH-meter probe (Hanna Instruments 211) into the mixture. Subsequently, the second aliquot was decarbonated and rinsed several times. Wet sieving was then performed in order to collect the 50–200 μm grain size fraction. This fraction was dried in the oven at 40°C and weighed. Heavy and light minerals were separated using bromoform at 2.89 g/cm^3^ following the Duplaix method [77]. Each fraction was weighed. The heavy minerals were put into Canada balsam and the mineralogy as well as number of grains (>100 grains) were determined with a polarized microscope. The light fraction was used for the analysis of the quartz grain morphoscopy. The morphoscopic class was determined from the degree of rounding and the surface aspect of at least 100 grains.

Mineralogical analyses were performed by XRD on powders of bulk samples. Analysis was conducted at the Department of “Histoire de la Terre” of the MNHN (UMR 7207 CR2P) on a D2 Phaser Bruker diffractometer equipped with a copper anode source (Cu Kα radiation). After the mineralogical identification, semi-quantitative estimates of mineral percentages were calculated with Diffrac.Eva software.

## Appendix 2: OSL, protocols and comments

### Equivalent Doses

#### Sample preparation

Samples were dried and sieved with a 2 mm mesh. The < 2 mm fraction was then split into two parts. One was used for the determination of the U, Th, and K content, the second was used for the De determination. For this last one, extraction of 200–250 μm quartz grains was performed following a standard procedure: wet sieving, HCl 10% then H_2_O_2_ 30% for removal of carbonates and organic materials respectively, high density (2.58 and 2.72) solution of sodium heteropolytungstate for separation of quartz, feldspars and heavy minerals, and HF 40% etching for 60 min (followed by HCl) to remove the matter irradiated by alpha particles. Finally, an IRSL test [78] was performed to confirm the absence of any feldspar contamination. The quartz grains were mounted either on stainless steel discs with silicon oil (1 mm mask) or on single grain discs with 100 holes of 300 μm diameter and 300 μm depth each.

#### Measurements

All luminescence measurements were performed with a Risø TL/OSL-DA 15 reader equipped with a 10 mW Nd:YVO_4_ diode-pumped laser (532 nm) for single grain excitation [79–81] and with clusters of blue (470 ± 20 nm) or infra-red (870 ± 30 nm) LEDs for multi-grain excitation. The reproducibility of the green laser excitation was estimated to be 1.9 to 2.7% and the reproducibility of the blue LED was 1.5%. Detection in the UV range (280–380 nm) was performed with a Q9235 Photomultiplier tube preceded by 7.5 mm of Hoya U340 filter. Irradiations were performed with a ^90^Sr/^90^Y beta source delivering 0.16 Gy/s to the 200–250 μm quartz grains.

#### Protocol and analyses

**Fig 11:**
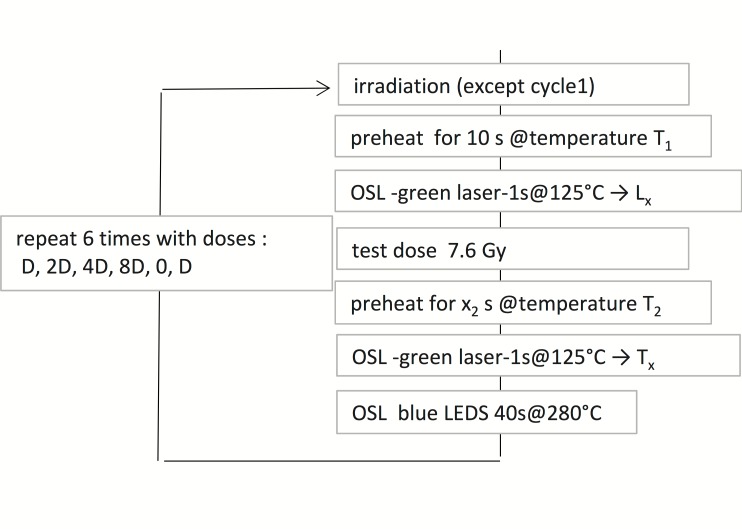
SAR protocol used for the single-grain De determination. The preheat parameters were fixed for each sample according to the results of the dose recovery tests (Table 6). For the multi-grain De determination, the protocol is the same, but for the excitation, performed with blue LED for 40 s.

The single-grain SAR protocol is presented in Fig 11. Analyses were performed with Analyst V.3.24 [82]. Exponential plus linear functions were used for curve fitting. For multi-grain measurements, the blue LEDs were stimulated for 40s; the first 0.8 s and last 4.16 s were used for signal and background respectively. Similarly the first 0.06 s and last 0.12 s (of 1 s of stimulation) were used for signal and background in single grain measurements, respectively.

**Fig 12:**
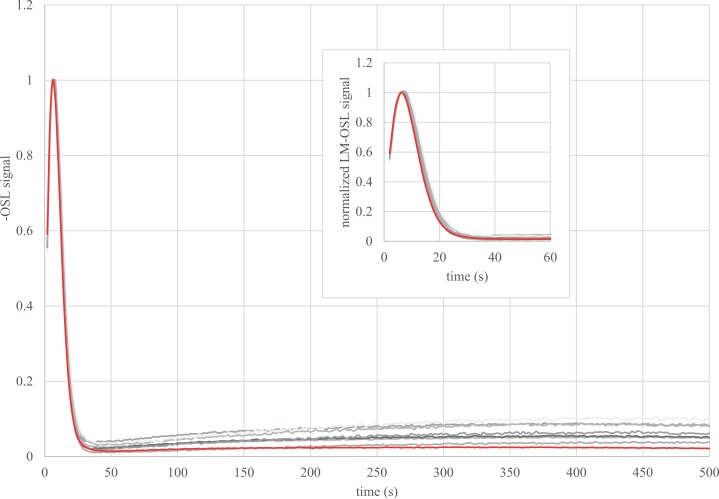
LM-OSL curves. For each sample a regenerative dose was given to a multi-grain aliquot. After a 260°C for 10 s preheat, the intensity of the blue LED was increased linearly from 0 to 50 mW/cm^2^ for 500 s. The curves are compared to the LM-OSL glow curve of the Risoe calibration quartz (in red), which is known to be dominated by a fast component.

The quartz samples from Buticha are remarkably bright and the single grain output is rather good (> 10% of the measured grains can be selected for the De determination). An example of glow and growth curve is presented in S2 Fig. It could be verified that the multi-grain signal is dominated by the fast component (Fig 12).

#### Testing the protocol

The appropriateness of the parameters for the SAR protocol were checked: dose recovery tests and preheat plateau tests were conducted. For the dose recovery test, three multi-grain aliquots of each sample were bleached (1 hour in a solar simulator), given a known dose close to the grossly estimated equivalent dose (De) and the SAR protocol was applied. The unweighted arithmetic means are compared to the expected dose (S3 Fig). The dose recovery ratios were found to be consistent with unity at two sigma or within ±10% of unity for a test dose preheat of 220°C for 10 s for GDB 4, 5, 6 and 7, 160°C cut-heat for GDB 1, 3 and 9 and 240°C cut-heat for GDB8 (Table 6A). GDB2 was not tested any further.

**Table 6:** Results of the tests for laboratory given doses.

a																
irradiation type	sample GDB	preheat parameters for…		given dose (Gy)	multigrain			single grain								
		natural and regenerative dose	test dose		n	ratio		n	overdispersion (%)		ratio—CAM		ratio- unweighted mean		ratio -bayesian estimates	
beta	9	260 10s	160 cut	6	3	1.01	±0.02	91/200	11	±1	1.04	±0.01	1.04	±0.02	1.07	±0.01
	8	260 10s	240 cut	6	3	1.00	±0.02	nd	nd		nd		nd		nd	
	7	260 10s	220 10s	9	3	0.98	±0.02	26/200	8	±2	1.04	±0.02	1.07	±0.03	1.08	±0.03
	6	260 10s	220 10s	9	3	0.99	±0.03	60/300	9	±2	1.00	±0.02	1.01	±0.02	1.06	±0.02
	5	260 10s	220 10s	18	3	0.99	±0.02	64/300	14	±2	0.99	±0.02	1.01	±0.02	1.05	±0.02
	4	260 10s	220 10s	55	3	0.98	±0.03	54/200	16	±3	0.95	±0.03	1.00	±0.03	0.98	±0.03
	3	260 10s	160 cut	74	3	0.96	±0.04	44/200	20	±3	0.95	±0.04	1.03	±0.06	1.03	±0.04
	1	260 10s	160 cut	111	3	0.96	±0.05	25/300	22	±5	1.01	±0.06	1.22	±0.16	1.04	±0.07
b																
gamma	8	260 10s	240 cut	9				228/700	19	±1						

a) dose recovery tests: the samples were bleached in a solar simulator and given a known beta dose in the OSL reader; b) Minimal intrinsic OD estimate: the sample was bleached in a solar simulator and given a known gamma dose outside the OSL reader. The number “n” of multi-grain aliquots or of selected over measured single grain is indicated, as well as the ratio of estimated to given beta dose. The ratio are given following the central De model (CAM) of [55], the Bayesian model of [57], or the arithmetic mean advocated by [56].

A second set of dose recovery tests was then performed with single grain discs using the parameters previously selected. Rejection criteria, based on the recycling ratio (within 0.9–1.1), signal to noise ratio (> 3), precision of the natural test dose signal (RSD > 10%), and recuperation (sensitivity corrected signal for a 0 dose > 5% of the sensitivity corrected natural signal) were used. The grains were also rejected when there was no intercept of the natural signal with the growth curve, or in case of intercept with infinite error estimate (saturation). Then, the dose was estimated either using an unweighted mean [56], or the central dose model (CAM, [55]), or the Bayesian SAR model [57]. The efficiency of the SAR parameters was confirmed (Table 6A).

Preheat plateau tests were performed using multi-grain discs for the samples whose De distributions were the least dispersed, i.e. GDB 4, 6, 7, 8 and 9. The preheat of the natural or regenerative dose was set at 220, 240, 260 or 280°C for 10 s (4 to 6 discs for each temperature) while the preheat of the test dose was chosen according to the results of the dose recovery tests. No significant dependence of the De on the preheat temperature was observable (S4 Fig). Therefore, we used a 260°C preheat for De determination for each of the GBD samples in this study (Table 4).

#### Are the De distributions consistent with a single dose component?

The sedimentological analyses suggest that the fine sands and silts, especially those of Complex II, have an aeolian origin (see §5.1). Moreover, the samples were collected close to the cave entrance where the sediments are exposed to sunlight. The quartz grains are clean, i.e. there is no embedded crust that would prevent the light from going through. Therefore, poor bleaching seems unlikely. As for the likelihood of bioturbation, burrows were observed in the section but the OSL sampling was cautiously conducted away from those. Burrowing by insects, however, might be more difficult to detect.

Some researchers have also suggested that the consistency between the multi-grain De and the single-grain De could be used as an evidence for single component (e.g. [83]). The multi-grain CAM De (mostly from the preheat plateau tests) (S3 Table) and the single-grain CAM De (Table 4) are consistent at one or two sigma for all samples except for GDB9 (3 sigma) and GDB1. For GDB1 and GDB3 however, the number of multi-grain measurements is low (n = 6 and 5 respectively) and the calculated CAM De_s_ might not be significant because of the scatter in these samples.

Besides these observations, the OD is considered as a key parameter for interpreting the De distribution, as it should reflect the story of the sample: it depends on intrinsic factors (e.g., how well each grain responds to the protocol) as well as extrinsic factors (bleaching before and bioturbation after deposition, as well as heterogeneity of the Dr at the millimeter/micrometer scale). However, there is no typical value of OD for well-bleached undisturbed samples (e.g., [84, 85]). More particularly, it has been shown from laboratory-dosed samples that the OD 1) increases with the central (or mean) De, 2) is higher for samples dosed outside the OSL reader than for those directly dosed inside it [85, 86]. It was also shown with modeling and simulations that 3) the OD increases when–at constant mean radioactive content–the proportion of radioactive hotspots (e.g., coarse potassium feldspar grains) increases. High heterogeneity (i.e. high proportion of hotspots) generally occurs with low radioactive contents (e.g. a few feldspar grains in a quartz sand dune) [84, 87]. The OD for the artificially dosed grains and most of the natural samples of Goda Buticha follow these patterns (Tables 4 and 6, and Fig 9). This statement, detailed in the following sentences (1-2-3), is based on the assumption that the OD of the different samples from Goda Buticha can be compared to each other. The samples have indeed similar geological origin, and similar LM-OSL glow curves. This assumption might have some limitations since, for example, different preheat parameters had be used (after dose recovery tests) and different artificial or natural doses are concerned. The patterns seem to be followed for three reasons: (1) The OD of the bleached and beta-dosed samples (dose recovery tests) increases from 8 ± 2 to 22 ± 5% when the given dose increases from 6 to 111 Gy. (2) One sample has been bleached and gamma dosed (GDB8). Its OD is 19 ± 1% for 9 Gy, to be compared to 10% for about 10 beta-derived Gy for other samples (Table 6B). This value gives the minimum OD to be expected for the natural samples with doses over 10 Gy. (3) The natural OD for GDB 6, 7 and 8 are only slightly higher (20 ± 2 to 26 ± 4%), suggesting that the supplementary OD due to external factors is weak. This can be expected from the very high Dr (i.e., K content) for these samples (Table 3). It is therefore reasonable to consider that these De distributions are consistent with a single De component. The increase of OD for GDB 4, 3 and 1 could be related to the increase of the CAM De, though it is difficult to ascertain that no other factors interfere. Meanwhile, we have also used a single-dose model for these samples. GDB5 and 9 have higher OD compared to what could be expected from the mean or CAM De and Dr for other samples. Whether this is due to the fact that these samples are not fully comparable to the others (e.g., different preheat parameters were used) or due to beta Dr heterogeneity is not conclusive. The Dr modeling that has been performed on GDB5 (Appendix 3) cannot help to answer this question because the model used is not of sufficient accuracy for that purpose (for example, the possible heterogeneities within the fine matrix were not taken into account). Only the comparison with the ^14^C data suggests *a posteriori* that the hypothesis of a single component distribution is in fact reasonable, at least for GDB5.

## Appendix 3: Further comments on the beta Dr

The calculation of the beta Dr is complex because all the sediment samples show heterogeneities at the millimeter scale, with a fine fraction (mainly < 1 mm) embedded in coarser material (bones, charcoals, calcareous crusts, different types of lithic fragments, etc.). Since the travel range for the beta particles is about 2 mm in rocks and sediments, the possible influence of this coarse material on the mean Dr received by the quartz grains requires special attention [51]. While the > 1 cm particles can be assumed to have no impact on the beta Dr because of their strong auto-absorption [87], particles in the thickness range between 2 mm and 1 cm require a separate analysis.

In the following discussion we must distinguish:

*D*_*fine*_: theoretical dose rate (i.e. energy received per unit of mass and time) in the quartz grains calculated from the radioisotope content of the fine (< 2 mm) fraction, following the infinite matrix assumption.

*D*_*total*_: theoretical dose rate in the quartz grains calculated from the radioisotope content of the fine and coarse fraction (< 1 cm) (which will be referred as total fraction), following the infinite matrix assumption.

*D*_*grains*_: the true dose rate in the quartz grains.

*Em*_*fine*_: the energy emitted per unit of time and per unit of mass by the fine fraction.

*Em*_*total*_: the energy emitted per unit of time and per unit of mass by the total fraction, equivalent to the infinite matrix dose rate [88] of these mixed fractions.

*D*_*fine*_ and *D*_*total*_ are calculated respectively using the equations:

$$D_{fine}=Em_{fine}*F_{size}*WF_{fine}$$

$$D_{total}=Em_{total}*F_{size}*WF_{total}$$

where *F*_*size*_ is an attenuation factor that depends on the grain size and has been estimated by Guérin et al. (48) and *WF*_*fine*_ and *WF*_*total*_ are the attenuation factors due to the dose rate part absorbed by the water content in the fine and total fractions respectively, (as defined by [89]).

As discussed in the main text, the coarse fraction (2 mm– 1 cm) generally has lower radioisotope content than the fine matrix (< 2 mm) (Table 2A and 2B). Therefore, the infinite matrix assumption should not apply. How far from the beta *D*_*fine*_ or *D*_*tota*l_ is the true beta *D*_*grains*_? Two effects have to be considered, i.e., auto-absorption (or size of the emitter) and dilution (or distance between emitter and dosimeter):

-1) the amount of energy absorbed in the coarse fraction is more important than the energy emitted within it [90]; this implies that the quartz grains in the matrix receive a lower amount of energy than if the fine matrix only was present, i.e. *D*_*fine*_ > *D*_*grains*_
-2) only a part of the quartz grains are affected by the presence of the coarse material as many are actually far from them. Using the *D*_*total*_ instead of *D*_*grains*_ leads to dilution of the radioisotope content of the fine fraction and affects (excessively) all the quartz grains; this implies *D*_*total*_ < *D*_*grains*_.

Therefore, *D*_*grain*s_ is bracketed by *D*_*fine*_ and *D*_*total*_.

**Fig 13:**
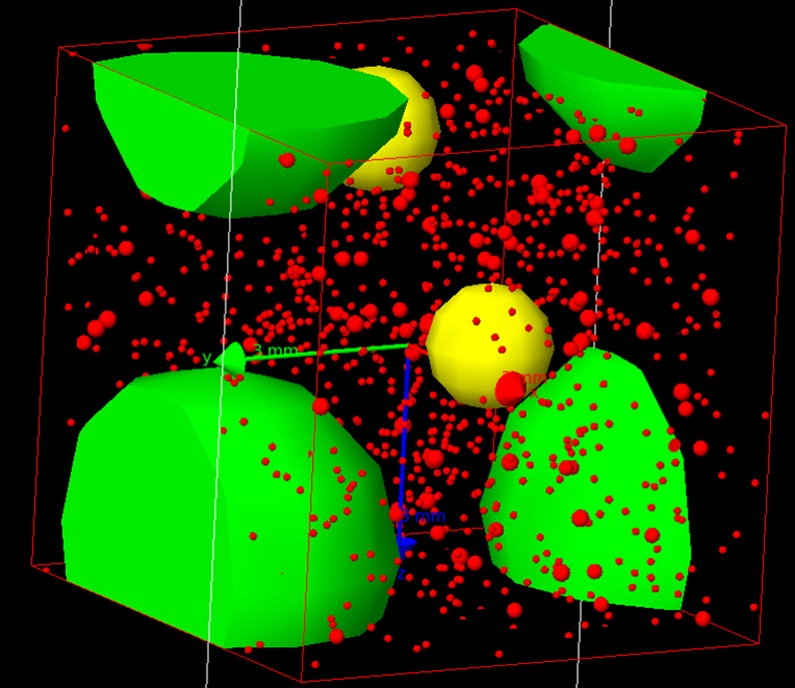
representation of the modelled sediment. The 5 mm diameter green spheres represent rock fragments, the 1.7 mm yellow spheres represent bones (with an identical auto-absorption of 2 cm long bones), the 100 to 300 μm red spheres represent a fraction of the quartz grains that are used as dosimeters, and the stacking box is a cube with a side length of 19.92 mm.

The calculation of *D*_*fine*_ and *D*_*total*_ for the samples shows that they are actually close to each other, despite the large difference in radioisotope contents. We have tested the predominance of one or the other of the above mentioned effects on the beta dose rate with a Geant4 simulation [51, 53, 91–93]. Sample GDB5 was chosen since it presents the largest difference between *D*_*fine*_ and *D*_*total*_. The fine fraction was modelled after its grain size histogram, chemical content and radioisotope contents; the coarse material was modelled considering 6.5% in weight of bones and 93.5% of stones, following weighting and sorting after dry sieving. The chemical composition of the bones was assumed and the chemical composition of the rocks was estimated using EDX-SEM analyses. Materials > 1 cm were not modelled since auto-absorption was assumed to dominate [90]. The different objects are represented by spheres of different sizes and chemical compositions, randomly located in a homogeneous medium, that represents the major part of the fine fraction (i.e. the matrix): the coarse fraction is represented by spheres of rocks of 5 mm diameter and spheres of bone material of 1.7 mm diameter (presenting the same beta auto-absorption than a 2 cm long bone) (Fig 13). Ten percent of the grains of the fine fraction between 100 and 300 μm diameter were also set as quartz spheres in the matrix. The remaining 90% of grains were not represented, as they have been taken into account in the matrix material composition. The quartz grains in the fine fraction are small enough in regards to the beta particle range that the effect on their Dr of the neighboring quartz grains can be ignored. The current water content of GDB5 is 6% (water/dry sediment). We have considered 3 values of moisture: 0% for a dry sediment, 6% in mass if the water is equally distributed in the fine and coarse fractions, 7.3% in mass if the water is in the fine fraction only.

**Fig 14:**
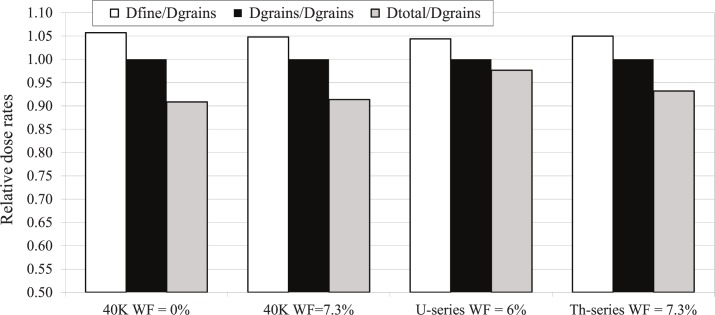
Comparison of dose rates from the simulation and from the infinite matrix assumption. White bars: *D*_*fine*_/*D*_*grains*_; grey bars: *D*_*total*_/*D*_*grain*_; black bars: *D*_*grains*_/*D*_*grains*_ (i.e. unity).

The emission of beta particles with spectra of ^40^K, U series and Th series from the coarse fraction or from fine fraction were simulated based on the measurement of U, Th, K contents in these fractions. The dose received in the quartz grains was recorded. Fig 14 presents the results for the 209 μm grain size. A comparison is made between the effective beta dose rates in the grains (*D*_*grains*_) in different conditions (with respect to radioactive element and moisture) and the dose rate calculated using the standard method applied to the fine or total fraction (*D*_*fine*_ and *D*_*total*_). The dose rates are normalized to *D*_*grains*_ in order to simplify the reading of the graph and to be independent of the simulation time.

Note that neither *D*_*fine*_ nor *D*_*total*_ seems to predominate in the effective *D*_*grains*_, whatever the moisture and the radioactive element considered. Note also that the merit of this modelling is to allow a relative comparison of the various estimates of the beta dose rate, although their simulated values cannot be directly used for the age calculation due to the rather rough representation of the model.

## Supporting Information

S1 Table Semi-quantitative results of XRD analyses for the Goda Buticha sediment samples. −: absent; ++: 1% < x ≤ 5%; +: ≤ 1%. The percentages are relative to the peaks heights in the diffractogram.
(PDF)

S2 Table Activities for the < 2 mm fraction. The ratios allowing comparison of the activities of the top, middle and bottom of the ^238^U decay chain are indicated.
(PDF)

S3 Table Equivalents doses for the multi-grain aliquots. The test dose preheats for GDB8 are 20°C lower than the corresponding regenerative dose preheat. N corresponds to the number of measured and accepted aliquots.
(PDF)

S1 Fig Photo of the section of the Goda Buticha sedimentary sequence in 2008. (JPG)

S2 Fig Example of glow (a) and growth curve (b) for one sample (GDB4, multi-grain aliquot) of Goda Buticha.
(PDF)

S3 Fig Results of various dose recovery tests for multi-grain aliquots. The test dose preheats were either 160°C cut (orange dots), or 220°C for 10 s (blue dots), or 240°C cut (grey dots). Each point is the arithmetic mean of three estimates.
(PDF)

S4 Fig Preheat plateaus. **Each dot represents the De for one measured multi-grain aliquot.** Preheat parameters, number of aliquots and central (CAM) De_s_ can be found in S3 Table.
(JPG)
