# Supplementary material for: Across the Gap: Geochronological and Sedimentological Analyses from the Late Pleistocene-Holocene Sequence of Goda Buticha, Southeastern Ethiopia
Source: PLoS One. 2017 Jan 26;12(1):e0169418. doi: 10.1371/journal.pone.0169418 (PMC5268652; doi:10.1371/journal.pone.0169418)
Supplement: S1 Table — −: absent; ++: 1% < x ≤ 5%; +: ≤ 1%. The percentages are relative to the peaks heights in the diffractogram. (PDF) [file pone.0169418.s001.pdf]

Stratigraphy				Magmatic rock minerals					Micas		Clays		Carbonates		Phosphates			Evaporites				Ca-oxalates	
Complex	Layers	Samples	Z (cm)	Hornblende	Quartz	Orthoclase	Plagioclases	Rutile	Muscovite	Chlorite	Kaolinite	Montmorillonite	Calcite	Dolomite	Carbonate-apatite	Dittmarite	Whitlockite	Gypsum	Anhydrite	Halite	Sylvite	Weddellite	Whewellite
Complex I	Ia3	S2	210.6	-	24.1	6.8	23.9	-	13.4	++	-	-	9.3	-	++	-	++	-	7.6	++	+	-	-
	Id	OSL9	191.6	++	9.7	7.6	21.3	-	14	-	-	-	15.6	-	++	-	++	-	16.1	++	+	-	-
	Ie	S3	188.5	-	10	7.7	25.3	-	11.5	-	-	-	13	++	6.3	-	5.8	6.9	8.3	++	+	-	-
	If	S6	189.4	-	12.7	-	-	-	-	-	-	-	75.8	-	++	-	5.7	-	-	++	+	-	-
		OSL8	188.2	-	9	++	-	-	7	-	-	-	64.6	-	++	8.6	++	-	-	+	+	-	-
	Ig	S4	181.5	-	9.5	5.7	32.5	-	-	-	-	-	30.6	-	7.7	-	5.3	-	-	++	6.5	-	-
	Ih	S8	182.5	-	5.9	6.9	23.9	-	10	++	-	-	++	-	++	19.1	++	-	-	++	+	++	15.3
Complex II	IIa	OSL7	172.5	5.6	20.6	-	22.8	-	10.7	++	-	-	21.5	-	++	-	-	-	-	+	+	++	8.4
	IIb	OSL6	166	5.2	16.4	-	33.7	-	10.1	-	-	-	21.3	-	++	-	-	-	-	+	+	++	6.1
		S9	165.9	++	22.1	-	28.9	-	10	-	-	++	11.7	-	++	++	++	-	-	+	++	++	5.9
	IIc	S10	156.9	-	12.3	-	21.3	-	-	-	-	-	15.9	-	9.6	12	-	-	-	++	+	14.5	12.3
		OSL5	145.9	6.1	22.4	-	23.1	-	12.2	-	-	-	29.7	-	++	-	-	-	-	++	+	-	-
		S12	141.5	6.3	13.6	5.3	32.8	-	11.3	++	++	-	17.9	-	++	-	-	++	-	+	+	-	-
		S11	129.7	-	25	++	18.8	-	9.2	-	-	-	14.8	-	++	-	6.9	17.7	-	+	+	-	-
		S14	115.5	++	9.3	++	21.4	-	10.9	++	++	-	10.6	-	++	-	-	30.6	-	+	+	-	-
		S13	112.5	-	13	-	23.5	-	9.9	++	++	-	29.3	-	++	-	-	15.6	-	+	+	-	-
		OSL4	104.3	++	8.9	-	27.3	-	11.1	-	-	-	++	-	++	-	-	39.8	-	+	+	-	-
	IId	S15	86.8	-	15	++	21.7	-	10	-	++	++	25	-	-	-	-	17.3	-	+	+	-	-
		S20	46.6	-	7.7	7.6	17.4	-	9.7	-	++	-	20.3	-	-	-	-	40.6	-	+	+	-	-
	IIe	S17	73.2	-	17.2	6.8	29.3	-	13.8	-	-	++	++	-	9.9	-	8.7	11.6	-	++	+	-	-
		OSL3	67.6	++	13	7.7	14.9	-	11.5	-	-	-	++	-	++	-	-	49.7	-	+	+	-	-
		OSL2	41.3	-	9.2	-	15.2	++	12.1	-	++	-	6.5	-	5.2	-	-	44.3	-	+	+	-	-
	IIf	S19	35.3	++	12.5	-	27.5	-	11.4	++	-	-	12.7	-	++	-	8.3	17.3	-	+	+	-	-
		S21	0.8	-	13	5.7	35.3	-	8.7	++	++	-	11.4	-	++	-	-	23.3	-	+	+	-	-
		OSL1	0	-	13.5	6.9	15.6	-	11.4	++	-	-	15.8	-	++	-	-	37.9	-	+	+	-	-

Table S1
