# Supplementary material for: Across the Gap: Geochronological and Sedimentological Analyses from the Late Pleistocene-Holocene Sequence of Goda Buticha, Southeastern Ethiopia
Source: PLoS One. 2017 Jan 26;12(1):e0169418. doi: 10.1371/journal.pone.0169418 (PMC5268652; doi:10.1371/journal.pone.0169418)
Supplement: S2 Table — The ratios allowing comparison of the activities of the top, middle and bottom of the 238U decay chain are indicated. (PDF) [file pone.0169418.s002.pdf]

Sample GDB	activities (Bq/kg)					ratios for ^{238}U series	
	^{238}U series			^{232}Th series	^{40}K		
	(^{234}Th)	(^{226}Ra)	(^{210}Pb)			$^{234}Th/^{226}Ra$	$^{210}Pb/^{226}Ra$
9	15.80 ±1.26	20.28 ±0.40	14.42 ±2.01	9.03 ±0.36	2348.1 ±17.45	0.78 ±0.06	0.71 ±0.10
8	8.40 ±0.44	12.24 ±0.17	13.49 ±1.27	7.44 ±0.23	780.1 ±7.14	0.69 ±0.04	1.10 ±0.10
7	23.62 ±1.77	24.61 ±0.47	21.19 ±1.61	16.76 ±0.80	1090.3 ±9.00	0.96 ±0.07	0.86 ±0.07
6	27.88 ±2.68	23.84 ±0.57	20.54 ±2.02	18.07 ±1.10	947.5 ±9.63	1.17 ±0.12	0.86 ±0.09
5	27.78 ±2.02	19.92 ±0.34	15.70 ±1.45	16.30 ±0.76	849.2 ±7.49	1.39 ±0.10	0.79 ±0.07
4	25.37 ±1.94	20.71 ±0.38	14.48 ±1.49	16.69 ±0.82	900.7 ±8.12	1.23 ±0.10	0.70 ±0.07
3	17.29 ±1.05	12.89 ±0.18	10.94 ±1.25	11.89 ±0.45	621.6 ±6.03	1.34 ±0.08	0.85 ±0.10
2	16.00 ±0.82	13.08 ±0.16	10.03 ±1.07	10.42 ±0.33	525.6 ±4.97	1.22 ±0.06	0.77 ±0.08
1	13.23 ±0.81	13.67 ±0.22	12.55 ±1.32	10.37 ±0.40	542.9 ±6.05	0.97 ±0.06	0.92 ±0.10
