# Supplementary material for: Across the Gap: Geochronological and Sedimentological Analyses from the Late Pleistocene-Holocene Sequence of Goda Buticha, Southeastern Ethiopia
Source: PLoS One. 2017 Jan 26;12(1):e0169418. doi: 10.1371/journal.pone.0169418 (PMC5268652; doi:10.1371/journal.pone.0169418)
Supplement: S3 Table — The test dose preheats for GDB8 are 20°C lower than the corresponding regenerative dose preheat. N corresponds to the number of measured and accepted aliquots. (PDF) [file pone.0169418.s003.pdf]

sample GDB	preheat parameters		n	De (Gy)
	natural and regenerative dose	test dose		CAM
9	240 to 300°C 10 s	160 cut	24	4.5 ±0.2
8	220 to 280°C 10 s	200 to 260°C cut	25	5.7 ±0.5
7	240 to 300°C 10 s	220 10s	24	7.9 ±0.3
6	240 to 300°C 10 s	220 10s	26	9.9 ±0.9
4	240 to 300°C 10 s	220 10s	16	70.6 ±3.3
3	260°C 10s	160 cut	5	85.9 ±4.5
1	260°C 10s	160 cut	6	86.7 ±5.7

Table S3
