# Supplementary figures and images for: Across the Gap: Geochronological and Sedimentological Analyses from the Late Pleistocene-Holocene Sequence of Goda Buticha, Southeastern Ethiopia
Source: PLoS One. 2017 Jan 26;12(1):e0169418. doi: 10.1371/journal.pone.0169418 (PMC5268652; doi:10.1371/journal.pone.0169418)

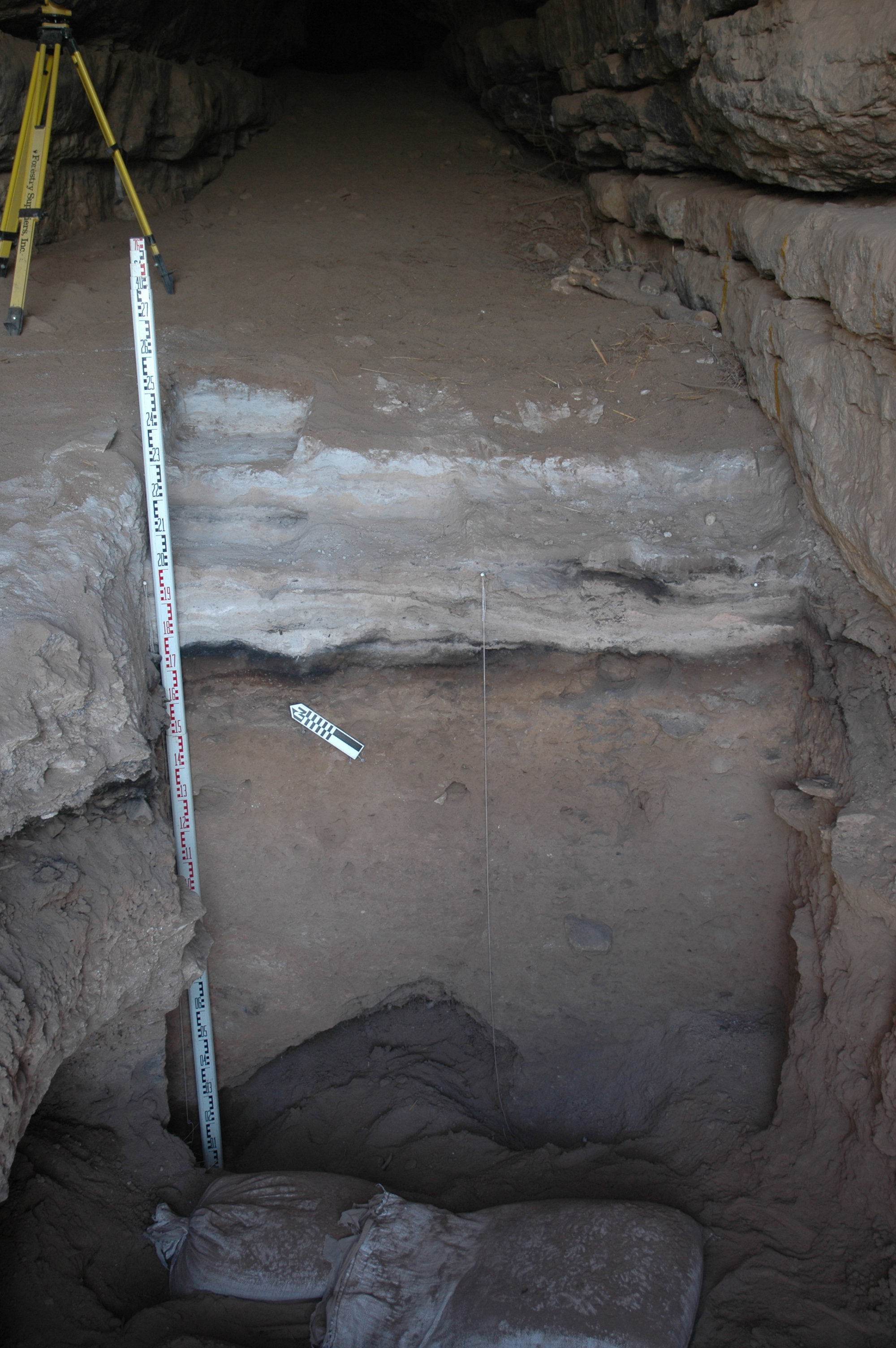

Supplement: S1 Fig — (JPG) [file pone.0169418.s004.jpg]

**a**

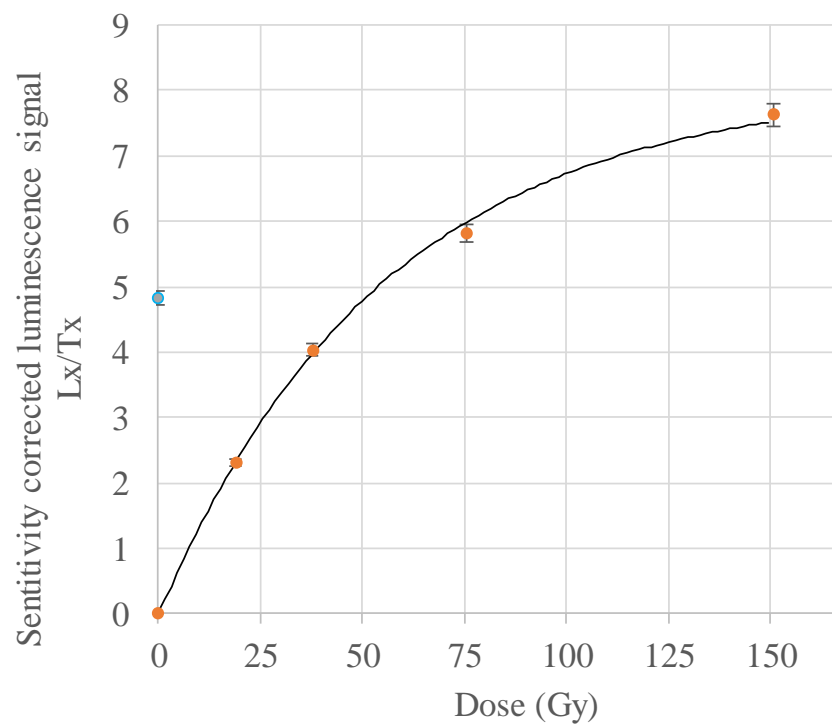

**b**

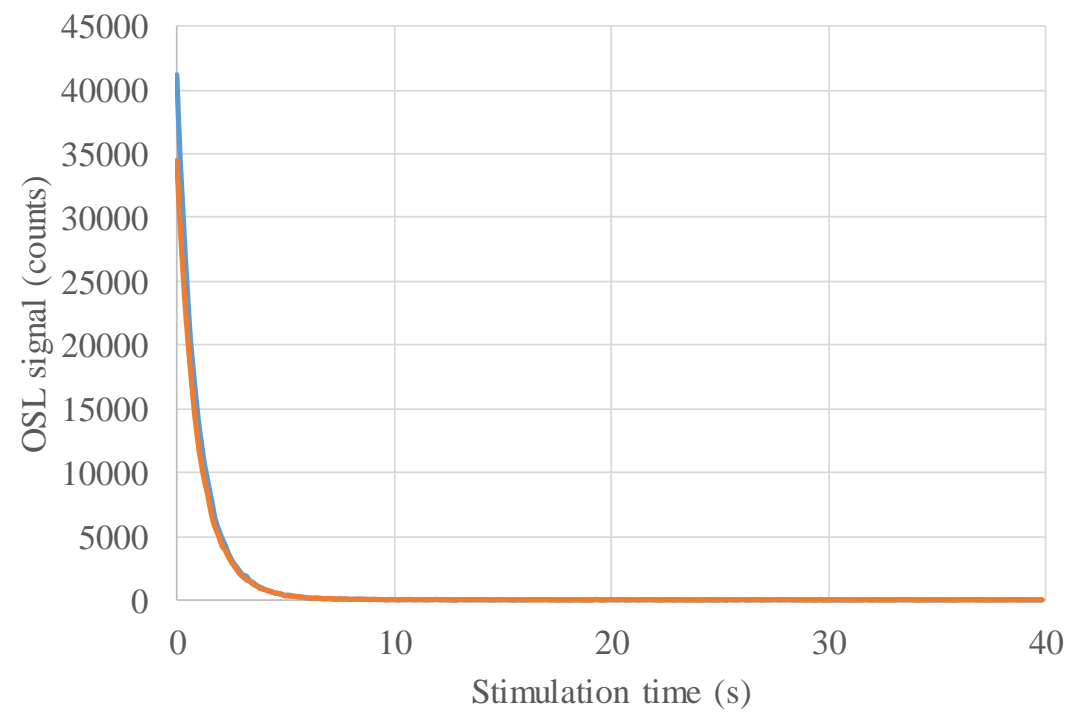

**Fig S2**

Supplement: S2 Fig — Example of glow (a) and growth curve (b) for one sample (GDB4, multi-grain aliquot) of Goda Buticha. (PDF) [file pone.0169418.s005.pdf]

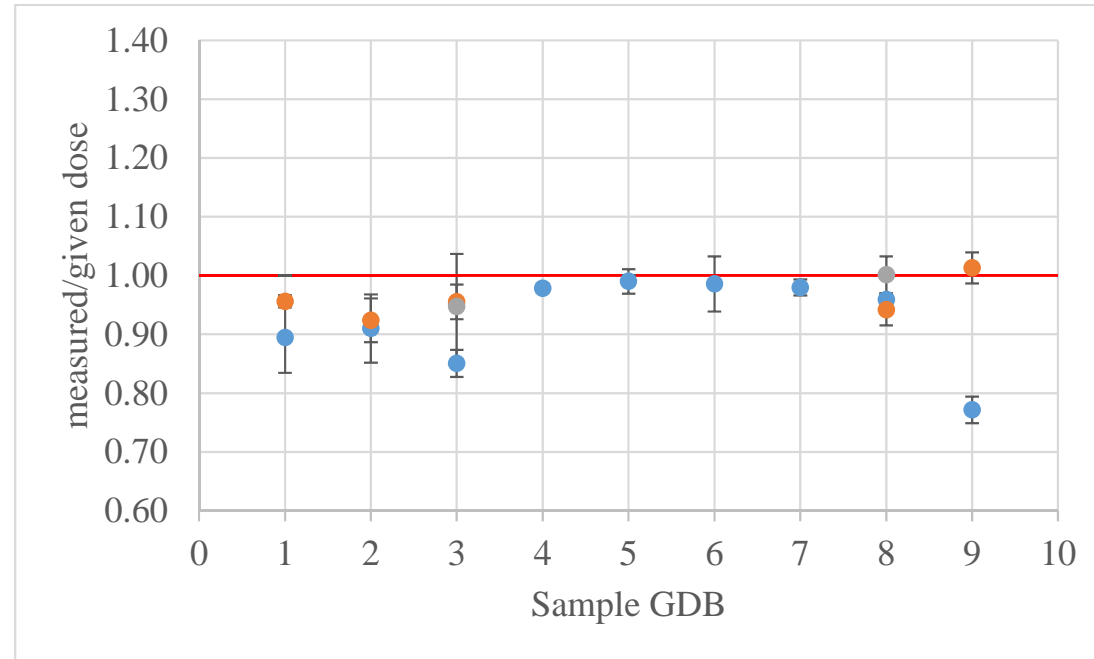

Fig S3

Supplement: S3 Fig — The test dose preheats were either 160°C cut (orange dots), or 220°C for 10 s (blue dots), or 240°C cut (grey dots). Each point is the arithmetic mean of three estimates. (PDF) [file pone.0169418.s006.pdf]

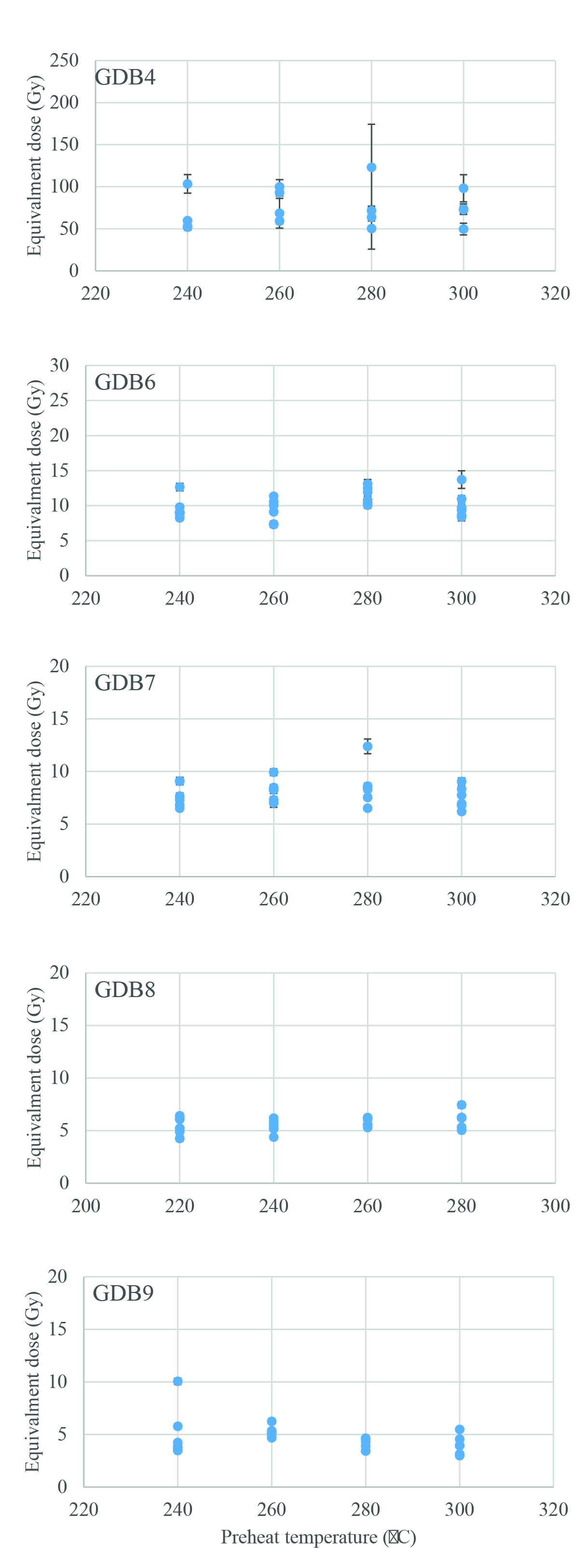

Supplement: S4 Fig — Each dot represents the De for one measured multi-grain aliquot. Preheat parameters, number of aliquots and central (CAM) Des can be found in S3 Table. (JPG) [file pone.0169418.s007.jpg]
